# Acupuncture combined therapy for the treatment of nape dorsal myofascial pain syndrome: meta-analysis, systematic evaluation, and GRADE evaluation

**DOI:** 10.3389/fmed.2025.1678696

**Published:** 2025-12-11

**Authors:** Yujing Zhang, Peizhen Zhang, Jingli Liu

**Affiliations:** College of Acupuncture and Massage, Changchun University of Traditional Chinese Medicine, Changchun, China

**Keywords:** acupuncture combined therapy, nape dorsal myofascial pain syndrome, randomized controlled trials, meta-analysis, GRADE evaluation, system evaluation

## Abstract

**Objective:**

This study aimed to assess the therapeutic efficacy and safety risks of acupuncture combined therapy in nape dorsal myofascial pain syndrome (MPS) management.

**Methods:**

Clinical randomized controlled trials of acupuncture combined therapy for nape dorsal myofascial pain syndrome were retrieved from databases such as China National Knowledge Infrastructure (CNKI), VIP Chinese Journal Platform, Wanfang Data, SinoMed, Web of Science, PubMed, Embase, and Scopus, covering publications from respective inception dates until 26 February 2025. Methodological quality appraisal involved dual assessments: GRADEprofiler 3.6 evaluated evidence certainty, while the Cochrane risk of bias (RoB) 2.0 tool analyzed bias risk. Statistical computations encompassed meta-analysis, heterogeneity analysis, sensitivity analysis, subgroup analysis, publication bias analysis, and trim-and-fill methods performed using StataSE 15, stateMP 18, and RevMan 5.4.

**Results:**

The systematic review incorporated 21 randomized controlled trials with a pooled cohort of 1,630 patients. ➀ Meta-analysis revealed that compared with control groups, acupuncture combined therapy can improve Visual Analogue Scale (VAS); score [standardized mean difference (SMD) = −1.51, 95% confidence interval (CI) (−2.12, −0.90), *p* < 0.00001], clinical effective rate [relative risk (RR) = 1.15, 95%CI (1.1, 1.2), *p* < 0.00001], Range of Motion (ROM); flexion [MD = 7.76, 95%CI (0.64, 14.88), *p* = 0.03], Pain Rating Index (PRI) score [MD = −0.45, 95%CI (−0.52, −0.38), *p* < 0.00001], and Oswestry score [MD = −0.30, 95%CI (−0.59, −0.01), *p* = 0.05]. ➁ Subgroup analysis indicated that acupuncture combined therapy demonstrated greater efficacy in pain reduction and cervical dysfunction improvement for patients with a mean age>35 years. ➂ Publication bias analysis identified potential bias for the VAS score and clinical effective rate, which may affect the reliability of VAS conclusions but not the clinical effective rate.

**Conclusion:**

Acupuncture combined therapy demonstrates clinically significant short-term benefits for nape dorsal MPS in pain relief and clinical effective rate. Existing limited evidence shows a low incidence of adverse events, but the risk profile cannot be fully clarified due to insufficient safety reporting in the majority of studies. Future rigorously designed, high-quality studies with diverse populations are needed to verify these findings.

**Systematic review registration:**

https://www.crd.york.ac.uk/prospero/, Unique Identifier: CRD420251000444.

## 1 Introduction

Myofascial pain syndrome (MPS) may result from injury, chronic strain, or idiopathic origins and is characterized by physical pain, movement disorders, muscle fatigue, and related symptoms. It is characterized by myofascial trigger points (TrPs) accompanied by muscle bandages, allergic points, and referred pain (1, 2). These trigger points are the cause of the patient’s pain complaint in clinical practice. Clinically, the diagnosis can be made by palpation to identify trigger points such as sensitive nodules and tight bandages (3). Therefore, the trigger point is the key to understanding, diagnosing, and treating myofascial pain syndrome. Patients with chronic non-specific neck pain are frequently affected by MPS. The most affected muscles of MPS are the superior trapezius, levator scapulae, etc. (4). Studies indicate that TrPs develop and progress following muscle overuse or direct injury, with potential mechanisms including eccentric overload, submaximal sustained contractions, and submaximal concentric contractions (5). Population-based studies estimate the prevalence of myofascial pain syndrome to be approximately 85%, with a significant predilection for females (6). At present, various interventions have been taken to treat myofascial pain syndrome in clinical practice (7). Drug therapy is a common clinical intervention that plays a role by affecting the mechanism of the nervous system. However, the use of drug therapy needs to consider the adverse effects on patients. For example, non-steroidal anti-inflammatory drugs (NSAIDs) can cause gastrointestinal discomfort and increase the risk of bleeding. Muscle relaxants cause drowsiness and dizziness in patients, while local analgesics cause elevated blood pressure and nausea in patients, cause allergic reactions in patients, and stimulate the skin (8).

Traditional Chinese medicine often uses palpation to find Ashi points for diagnosis and treatment. Ashi points are described as pain points or tenderness points on the patient’s body surface during palpation (9). Similar to the diagnosis of trigger points, it is also commonly used in the treatment of MPS. Acupuncture can stimulate nerve fibers and release endorphins, regulating pain signals to promote body recovery (8). Studies have shown that dry needling applied to TrPs, especially the acupuncture combined therapy, can effectively alleviate myofascial pain. This suggests that acupuncture combined therapy may produce a synergistic effect (8, 10). This study aimed to evaluate the efficacy and safety of acupuncture combined therapy in the treatment of nape dorsal MPS.

## 2 Data and methods

### 2.1 Research registration

Prospective registration was completed in the International Prospective Register of Systematic Reviews (PROSPERO) database (ID CRD420251000444), adhering to Preferred Reporting Items for Systematic Reviews and Meta-Analyses (PRISMA) 2020 guidelines for systematic reviews and meta-analyses (11).

### 2.2 Search strategy

The two evaluators searched relevant literature in databases such as CNKI, VIP Chinese Journal Platform, Wanfang Data, SinoMed, Web of Science, PubMed, Embase, and Scopus. Comprehensive searches covered all available data from source inception until 26 February 2025. The search was restricted to publications in Chinese and English due to the research team’s language capabilities and resource constraints for accurate translation and assessment. To ensure exhaustive literature coverage, the search strategy used Medical Subject Headings (MeSH) and keyword-based queries across targeted repositories, deliberately omitting geographical, linguistic, or regional restrictions. Database interrogation used the following core search terms: (‘acupuncture therapy’ OR ‘acupuncture’ OR ‘combine acupuncture and moxibustion’ OR ‘electroacupuncture’ OR ‘needling’) AND (‘nape dorsal myofascial pain syndrome’ OR ‘neck back myofascial pain syndrome’ OR ‘nape muscular fasciae inflammation’ OR ‘nape muscle fasciitis’ OR ‘nape back fasciitis’ OR ‘cervical back muscle fasciitis’ OR ‘myofascial pain syndrome’ OR ‘myofascial syndrome’ OR ‘back muscle fasciitis’ OR ‘fasciitis’ OR ‘dorsal shoulder fasciitis’ OR ‘cervical dorsal myofascitis’ OR ‘myofascial trigger point pain’ OR ‘myofascitis’ OR ‘Myofascial pain’) AND (‘randomized controlled trial’ OR ‘RCT’, ‘Randomized’ OR ‘clinical trial’ OR ‘clinical study’).

### 2.3 Inclusion criteria

① Research type: Randomized controlled trials of acupuncture combined therapy in the treatment of nape dorsal MPS. ② Subjects: Patients diagnosed with nape dorsal MPS based on authoritative diagnostic criteria (3, 12–14). The inclusion criteria permitted broad heterogeneity in baseline profiles, with no prespecified constraints on age ranges, sex distribution, and ethnic diversity. ③ Intervention measures: The intervention for the treatment group was acupuncture combined therapy. “Acupuncture combined therapy” refers to needle insertion (manual, electrical, or dry-needling) at acupoints, Ashi points, or trigger points plus at least one adjunct such as moxibustion, cupping, or massage. The rationale for combining modalities rests on the traditional Chinese medicine principle of “synergistic qi–blood regulation” and has been empirically tested in several RCTs; the control patients received non-acupuncture combined therapy, including simple acupuncture therapy, drug therapy, and exercise therapy. ④ Language restriction: Inclusion of literature in Chinese or English language. ⑤ Outcome indicators: VAS score, clinical effective rate, Neck Disability Index (NDI); score, cervical function ROM score, PRI score, Present Pain Intensity (PPI); score, SF-36 (The MOS Item Short Form Health Survey) score, Oswestry, Pressure Pain Threshold (PPT); score, and rest pain efficacy. The included literature outcome indicators should include one or more of them.

### 2.4 Exclusion criteria

① Non-randomized controlled studies, such as animal experiments, reviews, semi-randomized controlled trials, case reports, conference papers, medical records, and cohort studies. ② Repetitively published literature. ③ Cannot get the full text of the literature. ④ Interventions: Non-acupuncture combined therapy. ⑤ The experimental design is not rigorous, and the baseline is not comparable.

### 2.5 Literature screening

Two researchers (YJ. Z and PZ. Z) independently included and excluded the literature by reading the titles, keywords, and abstracts, excluding unrelated, repetitive literature. After cross-checking, the two researchers downloaded and read the full text. The literature screening process implemented predetermined inclusion/exclusion criteria to finalize selected studies, with systematic documentation of exclusion rationales for all discarded records. If the results of the two researchers differ, a third party is sought to assist in the ruling, and the final result is determined through discussion and consultation.

### 2.6 Data extraction

The data were extracted in the form of tables. Two evaluators independently extracted data based on the pre-designed tables. The extracted contents included first author, publication year, sample size, age, intervention measures, and outcome indicators.

### 2.7 Bias risk assessment

Bias risk stratification followed the Cochrane RoB 2.0 criteria across five core domains. Two researchers independently assigned risk classifications (low/unclear/high), consulting a third party for unresolved discrepancies after documented deliberation.

### 2.8 Evaluation of evidence quality

Two researchers independently used GRADE Profiler 3.6 to evaluate the quality of evidence. The evaluation content included risk of bias, discordance, indirectness, inaccuracy, and publication bias. According to the evaluation results, it was divided into high, medium, low, and extremely low. If there are objections during the evaluation process, a final decision will be made by a third party.

### 2.9 Statistical analysis

Statistical analyses were executed utilizing RevMan 5.4, Stata 18, and Stata 15 software. RevMan was used primarily for meta-analysis and the generation of forest plots. At the same time, Stata was used for additional statistical assessments, including funnel plot construction, meta-regression analysis, Egger’s and Begg’s tests, and the trim-and-fill analysis. Binary variables underwent the relative risk (RR) to calculate the effect size, while continuous variables used either mean difference (MD) or standardized mean difference (SMD), both of which took 95%CI. The statistical significance threshold was determined at a *p*-value of <0.05 (15). Heterogeneity assessments applied random-effects models when I^2^ > 50%; conversely, fixed-effects models were used below this benchmark. Potential heterogeneity sources, including variations in intervention protocols and time, were investigated through subgroup stratification and sensitivity. Sensitivity analysis verified the robustness of the combined results by eliminating each study one by one. Potential publication bias was evaluated by plotting funnel plots and combining Egger’s test and Begg’s test. The outcome indicators that may have publication bias were used to verify the robustness of the outcome using the trim-and-fill method. The significance threshold for publication bias was set at a *p*-value of <0.05 (16).

## 3 Results

### 3.1 Search results

**Figure 1:**
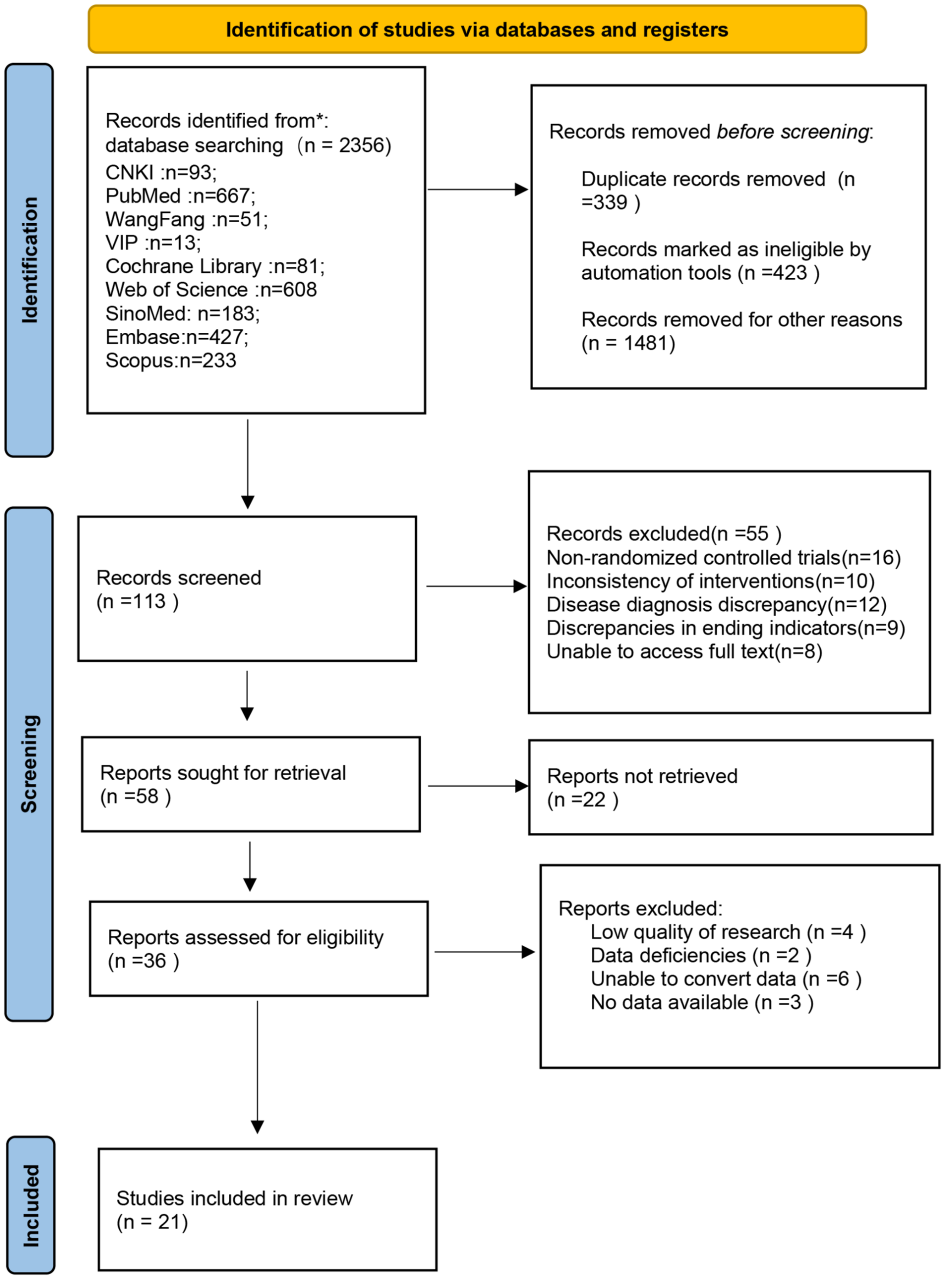
Screening process and results of literature.

The database interrogation yielded 2,356 articles. Repetitive literature, review literature, and case reports were excluded by reading titles and abstracts. A total of 21 articles were finally included based on predetermined inclusion and exclusion criteria. The screening process of the literature is shown in Figure 1.

### 3.2 Basic characteristics of literature

The 21 randomized controlled trials collectively enrolled 1,630 patients, with 819 individuals allocated to the therapeutic intervention group and 811 to the control arm. The sample size of the single group was 15–64 cases. Comparable baseline profiles were observed between the two groups. The literature included is primarily from Asian and Chinese databases, which reflects the geographic and cultural contexts in which acupuncture and moxibustion combination therapies are most widely practiced and researched, but there is also potential for geographic and selection bias. Among them, 18 outcome indicators included the VAS score (4, 17–33). A total of 12 studies involved the clinical effective rate (22–24, 26–34). Overall, eight studies involved the NDI score (4, 17, 19–21, 26, 31, 35); four studies involved the cervical function ROM score (4, 20, 21, 31); four studies involved the PRI score (18, 23, 29, 31); five studies involved the PPI score (18, 22, 23, 29, 31); three studies involved the SF-36 score (17, 25, 31); four studies involved the PPT score (4, 17, 18, 21); and three studies involved the Oswestry score (27, 28, 30). The detailed characteristics of the included literature are listed in Table 1.

**Table 1:** Basic characteristics of the included literature.

Literatures	Number of samples T/C	Course T/C	Mean duration T/C	Age T/C	Mean age T/C	Treatment group measure	Control group measures	Outcome indicator
Jing (33)	60/60	3 months ~ 10 years/2 months ~ 10 years	5.3 ± 0.5/5.3 ± 0.5 years	21 ~ 68/22 ~ 68	44.6 ± 7.5/45.1 ± 7.6	Self-made Chinese Herbal Hot Salt Plus Acupuncture	Acupuncture	①②
Wen-sheng et al (32)	34/34	1 ~ 24 months/1 ~ 24 months	13 ± 5.7/13 ± 5.7 months	24 ~ 58/24 ~ 58	42.12 ± 6.80/42.12 ± 6.80	Chest and back chiropractic manipulation combined with electroacupuncture	Electroacupuncture	①②
Xi-liang (31)	46/45	3 ~ 21 months/3 ~ 20 months	12.51 ± 2.75/12.01 ± 2.64 months	29 ~ 59/30 ~ 59	45.17 ± 5.09/45.87 ± 5.23	Acupuncture combined with indomethacin tablets combined with tizanidine hydrochloride tablets	Indomethacin tablets combined with tizanidine hydrochloride tablets	①②③④⑤⑥⑦⑧
Feng et al. (26)	32/30	—	431.8 ± 363.9/409.5 ± 352.8 days	—	47.26 ± 9.63/46.94 ± 11.56	Acupuncture of Ash-i points Combined with Moxibustion of Heat-sensitive Points	Acupuncture at Ash-i points with TDP irradiation	①②⑤⑥
Yan-hong et al. (27)	40/40	3 days ~18 weeks/2 days ~ 17 weeks	10.83 ± 4.12/11.27 ± 3.97 weeks	23 ~ 40/22 ~ 42	26.9 ± 5.8/27.1 ± 6.1	Ceramic Moxibustion Pot Cupping Combined with meridian tendon needling method	Meridian tendon needling method	①②㉗
Jing et al. (26)	47/47	—	5.51 ± 1.02/5.44 ± 0.95 months	27 ~ 60/26 ~ 59	40.82 ± 3.32/40.13 ± 3. 21	Heat-Sensitive Moxibustion Combined with Acupuncture	Acupuncture	①②③⑨⑩⑪⑫⑬⑭⑮⑯⑰⑱⑲⑳㉑㉒
Zhi-ping (34)	38/37	7 months ~ 21 years/9 months ~ 18 years	—	18 ~ 69/19 ~ 66	—	Triple acupuncture combined with cupping bleeding	Needle acupuncture combined with needle retention cupping	①
Bao-guo et al. (36)	60/55	4 months ~ 11 years/4 months ~ 11 years	6.3/6.3 years	14 ~ 65/14 ~ 65	27.6/27.6	Elongated needle combined with medicine-separated moxibustion	Western medicine therapy with infrared irradiation	㉓㉔
Yu-xia (25)	30/30	2 weeks ~ 3 months or more/2 weeks ~ 3 months or more	—	20 ~ 50/20 ~ 50	—	Jin three-needle combined with Mckenzie therapy combined with cupping	McKenzie therapy combined with cupping	①⑦㉕
Le-qin (24)	50/50	1 month ~ 6 years/10 days ~ 5 years	—	20 ~ 65/17 ~ 60	—	Scraping combined with warm acupuncture	acupuncture	①②㉖
Qi (23)	30/30	0.1 ~ 7 years/0.1 ~ 6 years	3.5 ± 0.4/3.3 ± 0.4 years	18 ~ 71/17 ~ 70	53.5 ± 12.8/53 ± 10.8	Electroacupuncture Combined with Point Injection	celecoxib capsule orally	①②⑤⑥
Ting (22)	50/50	2 ~ 18 months/3 ~ 15 months	4.45 ± 3.52/4.82 ± 3.33 months	22 ~ 73/25 ~ 74	52.28 ± 11.30/53.08 ± 10.52	Moxibustion combined with acupuncture	acupuncture	①②⑥
Li-hua (28)	34/34	3 ~ 20 years/2 ~ 20 years	12.31 ± 7.25/12.02 ± 8.19 years	20 ~ 71/26 ~ 73	53.36 ± 10.25/54.78 ± 10.95	Warm acupuncture combined with palm rubbing method	warm acupuncture	①②㉗
Jian and Hua (30)	43/43	1 ~ 12 years/1 ~ 11 years	7.3 ± 1.9/7.1 ± 1.8 years	36 ~ 68/35 ~ 70	57.9/58.2	Acupuncture of Jinggu (BL64) Combined with Massage	Massage	①②㉗㉘
Brennan et al. (35)	20/25	—	—	18 ~ 59/18 ~ 59	28 ± 9.99/26.32 ± 8.94	DN/IMES	DN	①㉙
Leon-Hernandez et al. (21)	29/30	—	19.36 ± 19.23/16.03 ± 17.23 months	—	25 ± 8/25 ± 8	DN + PENS	DN	①③④㉚㉛
Eftekharsadat et al. (17)	31/30	Over 2 months/Over 2 months	1.3 ± 0.7/2.0 ± 1.0 years	—	33.7 ± 5.8/23.3 ± 7.0	aerobic exercise +acupuncture	acupuncture	①③⑦㉚
Korkmaz et al. (19)	33/29	—	—	—	34.9 ± 7.3/34.2 ± 7.5	DNG	EG	①③㉜㉝
Sami Alattar and Alzahrani (20)	15/15	Over 3 months/Over 3 months	—	≥18/≥18	29.9 ± 4.6/29.5 ± 4.5	DN + usual physiotherapy	usual physiotherapy	①③④㉞
Jiang et al. (18)	33/33	—	2.9 ± 4.2/5 ± 6 months.	—	35 ± 7/37 ± 9	Effect of bloodletting therapy at local myofascial trigger points and acupuncture at Jiaji (EX-B 2) points	Lidocaine block	①⑤⑥㉚
Cerezo-Téllez et al. (4)	64/64	Over 6 months/over 6 months	—	—	48 ± 15.7/52 ± 16.6	DDN + passive stretching	passive stretching	①③④㉚㉟

T: treatment group; C: control group; —:not described; ① VAS score, ② clinical effective rates, ③ NDI score, ④ cervical function ROM score, ⑤ PRI score, ⑥ PPI score, ⑦ SF-36 score, ⑧ PSQI score, ⑨ interleukin-1β (IL-1β) content, ⑩ cyclooxygenase-2 (COX-2) content, ⑪ tumour necrosis factor-α (TNF-α) content, ⑫ 25-dihydroxy vitamin D [25(OH) D] content, ⑬ β-endorphin (β-EP) content, ⑭ 6-keto-prostaglandin E1α (6-keto-PGE1α) content, ⑮ substance P (SP) content, ⑯ 5-hydroxytryptamine (5-HT) content, ⑰ fascia thickness, ⑱ Tissue elasticity map score, ⑲ young’s modulus value, ⑳ average amplitude value, ㉑ average frequency slope, ㉒ GQOLI-74 score, ㉓ rest pain effect, ㉔ improvement of other symptoms, ㉕ Roland-Morris score, ㉖ pressure pain score, ㉗ Oswestry score, ㉘ The time taken to heal, ㉙ NPRS, ㉚ PPT score, ㉛ PNS, ㉜ thickness of UT muscle, ㉝ diameter of the MTrP, ㉞ BDI, and ㉟ neck muscles strength.

### 3.3 Evaluation of research quality

**Figure 2:**
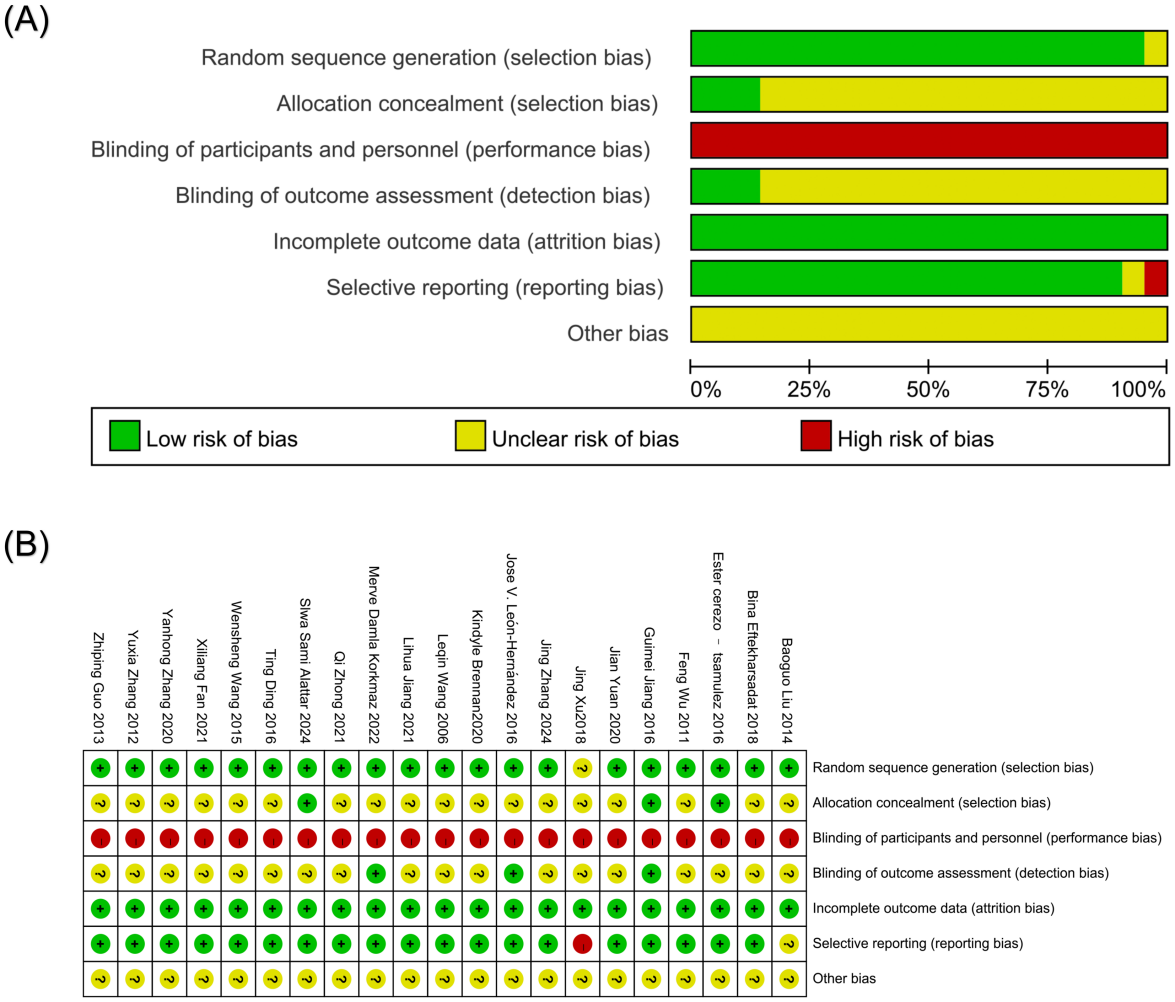
**(A)** Bias risk assessment of included literature; **(B)** Summary of bias risk assessment of included literature.

Regarding the bias of the randomization process, 20 studies were classified as low risk by the random number table method or randomization grouping (4, 17–32, 34–36). One study did not specify the randomization, so its random deviation risk could not be accurately assessed and was classified as unclear risk (33). Regarding allocation concealment, two studies used the envelope method (18, 20). Allocation concealment details were explicitly documented in one trial (4), warranting a low-risk classification; the remaining studies did not explain the details of the allocation of hidden, classified as unclear risk (17, 19, 21–36). In terms of blinding, due to methodological limitations inherent in the intervention, all studies were classified as high risk in blinding patients and operators; the three items were designed in a single-blind manner, so the blind rule of the evaluator of the research results was classified as low risk (18, 19, 21). The reports of the remaining studies lacked specific details on blinding and were assessed as unclear risks (4, 17, 20, 22–36). Regarding the data reporting of the results, all studies reported data completely and were identified as low risk. In terms of selective reporting bias, one study was classified as high risk because of shedding cases, and no reason was given (33). Five studies reported shedding cases and reasons for shedding and were rated as low risk (4, 17, 19, 21, 35); the rest of the studies were rated as low risk without shedding cases (18, 20, 22–32, 34, 36). Finally, in terms of other biases, all studies were identified as unclear risks due to insufficient information required for evaluation. The detailed information is shown in Figures 2A,B.

## 4 Meta-analysis of acupuncture combined therapy in the treatment of nape dorsal MPS

### 4.1 VAS score

**Figure 3:**
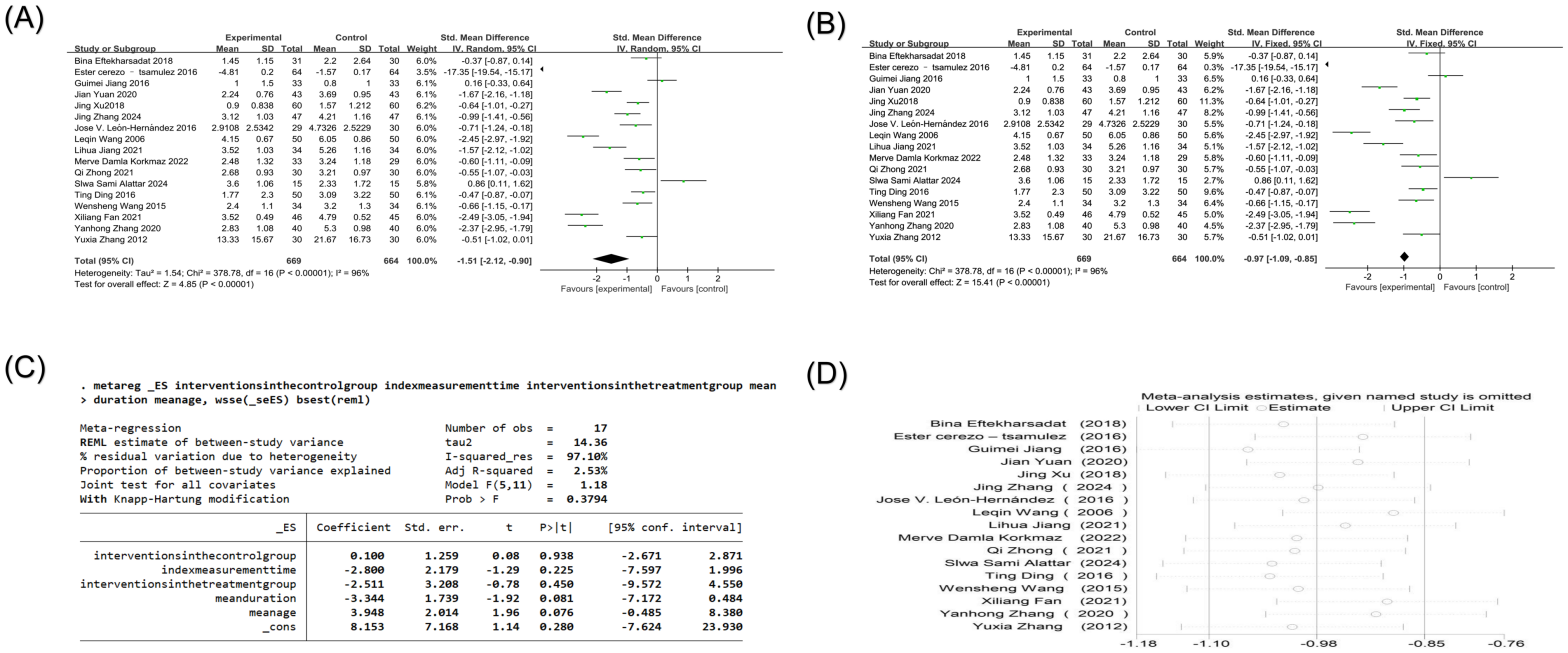
**(A)** Forest plot of VAS score of nape dorsal MPS treated by acupuncture combined therapy (random-effects model); **(B)** forest plot of VAS score of nape dorsal MPS treated by acupuncture combined therapy (fixed-effects model); **(C)** meta regression of VAS score of nape dorsal MPS treated with acupuncture combined therapy; **(D)** sensitivity analysis of VAS score of nape dorsal MPS treated with acupuncture combined therapy.

Eighteen studies reported VAS scores, one of which could not be converted, so the remaining 17 studies were analyzed (4, 17–28, 30–33). Since the units of VAS scores are not uniform, SMD analysis was used to eliminate the effect of measurement units on the meta-analysis results. Heterogeneity analysis showed high heterogeneity among studies (*p* < 0.00001, I^2^ = 96%). Given the substantial methodological and clinical diversity anticipated across studies, a random-effects model was used *a priori*, as it provides a more conservative estimate of the effect size and its precision when heterogeneity is present. The results of the random-effects model showed that the VAS score of the acupuncture combined group was significantly different from that of the control group [SMD = −1.51, 95%CI (−2.12, −0.90), Z = 4.85, *p* < 0.00001]. To assess the robustness of this finding, a sensitivity analysis was performed using a fixed-effects model, which yielded a similar point estimate but with a narrower confidence interval [SMD = −0.97, 95%CI (−1.09, −0.85), *p* < 0.00001], confirming the statistical significance of the pain reduction effect despite the high heterogeneity (Figures 3A,B). Due to the high heterogeneity, meta-regression analysis, sensitivity analysis, and subgroup analysis were performed. The results of the meta-regression analysis showed that covariates did not fully explain high heterogeneity. There was no significant effect on the control group measures, the treatment group measures, and the measurement time. The average course of disease (*p* = 0.081) was close to the mean age (*p* = 0.076), suggesting that the longer course of disease may reduce the effect size, and the older age may increase the effect size. However, due to the small sample size and insufficient model effectiveness, it is impossible to explain the source of heterogeneity effectively. It is necessary to consider other potential adjustment variables, including new variables, and further expand the sample size, perform subgroup analysis, and perform sensitivity analysis (Figure 3C). Sensitivity analysis showed that the robustness of the combined results was high, and the heterogeneity did not change significantly (Figure 3D). Potential sources of the extreme heterogeneity were further investigated. Clinically, variations may arise from differences in the specific acupuncture techniques used (e.g., dry needling, electroacupuncture, and manual acupuncture), treatment frequency and duration, co-interventions (e.g., moxibustion and cupping), and patient characteristics (e.g., baseline pain severity, chronicity, and presence of multiple trigger points). Methodologically, heterogeneity could be attributed to differences in VAS assessment protocols (e.g., 100 mm vs. 10 cm scales), blinding of outcome assessors, and study design (e.g., single-center vs. multicenter). Subgroup analysis identified that when the mean age was above or equal to 35 years, the effect of reducing VAS score in the treatment group was significantly better than that in the control group [SMD = −2.06, 95%CI (−2.96, −1.16), *P*<0.00001]. When the mean age was less than 35 years and the mean age of unknown patients, there was no significant difference in VAS scores between the two groups of patients [SMD = −0.65, 95%CI (−1.55, 0.24), *p* = 0.15] [SMD = −1.48, 95%CI (−3.38, 0.42), *p* = 0.13]. When the intervention in the control group was acupuncture or other treatments, the degree of pain reduction in the treatment group was significantly better than the control group [SMD = −1.13, 95%CI (−1.72, −0.53), *p* = 0.0002] [SMD = −2.17, 95%CI (−3.45, −0.90), *p* = 0.0009]. There was no significant difference in VAS score between the treatment group and the western medical control group [SMD = −1.52, 95%CI (−3.42, 0.38), Z = 1.56, *p* = 0.12]. In addition, the effect of reducing the VAS score of patients in the treatment group to relieve pain was not affected by the time of index measurement, the average course of disease, and the type of acupuncture combined therapy, which was significantly better than that of the control group (Table 2).

**Table 2:** Subgroup analysis of VAS score of nape dorsal MPS treated by acupuncture combined therapy.

Subgroup	Research quality	Sample size		Heterogeneity analysis		Meta-analysis		Effects models
		T	C	I^2^ (%)	*P*	SMD/MD/RR (95%CI)	*P*	
Interventions in the control group								
Acupuncture	7	307	307	91	<0.00001	−1.13 (−1.72, −0.53)	0.0002	Random
Western medicine	2	76	75	96	<0.00001	−1.52 (−3.42, 0.38)	0.12	Random
Other therapies	8	286	282	97	<0.00001	−2.17 (−3.45, −0.90)	0.0009	Random
Index measurement time								
<15 days	6	187	189	92	<0.00001	−0.87 (−1.66, −0.08)	0.03	Random
≥15 days	11	481	476	97	<0.00001	−1.96 (−2.81, −1.10)	<0.00001	Random
Interventions in the treatment group								
Acupuncture combined with moxibustion	3	137	137	93	<0.00001	−1.26 (−2.26, −0.25)	0.01	Random
Acupuncture combined with other therapies	14	532	527	96	<0.00001	−1.62 (−2.36, −0.88)	<0.00001	Random
Mean duration								
>34 months	4	167	167	83	0.005	−1.09 (−1.67, −0.52)	0.0002	Random
≤34 months	7	281	279	93	<0.00001	−1.02 (−1.70, −0.33)	0.004	Random
Not reported	6	221	218	98	<0.00001	−3.04 (−4.91, −1.16)	0.002	Random
Mean age								
≥35 years old	10	441	440	97	<0.00001	−2.06 (−2.96, −1.16)	<0.00001	Random
<35 years old	5	148	144	92	<0.00001	−0.65 (−1.55, 0.24)	0.15	Random
Not reported	2	80	80	96	<0.00001	−1.48 (−3.38, 0.42)	0.13	Random

T: treatment group; C: control group.

### 4.2 Clinical effective rate

**Figure 4:**
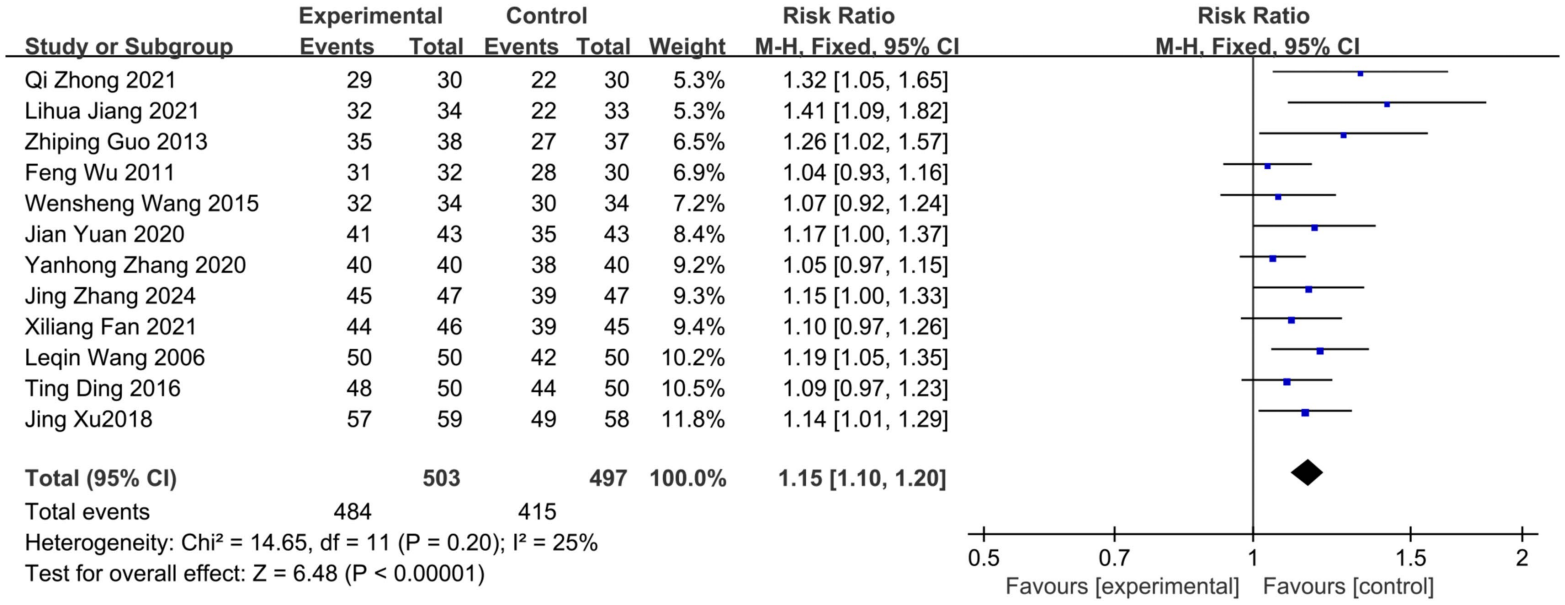
Forest plot of clinical effective rate of acupuncture combined therapy in the treatment of nape dorsal MPS.

A total of 12 literature outcome indicators included the clinical effective rate (22–24, 26–34). Heterogeneity analysis showed little statistical heterogeneity between the results of each study (*p* = 0.2, I^2^ = 25%). The results of the fixed-effects model showed that [RR = 1.15, 95%CI (1.1, 1.2), Z = 6.48, *P*<0.00001], and the clinical difference was statistically significant (*P*<0.01), indicating that the clinical effective rate of acupuncture combined therapy in the treatment of nape dorsal MPS was better than that of the control group, and acupuncture combined therapy had better efficacy (Figure 4). Subgroup analysis showed that the effect of the treatment group on improving the clinical effective rate of nape dorsal MPS was not affected by the type of intervention measures, the evaluation time of effective rate, the mean age, or the average course of disease of the treatment group and the control group, which were significantly better than those of the control group (Table 3).

**Table 3:** Subgroup analysis of clinical effective rate of acupuncture combined therapy in the treatment of nape dorsal MPS.

Subgroup	Research quality	Sample size		Heterogeneity analysis		Meta-analysis		Effects models
		T	C	I^2^ (%)	*P*	SMD/MD/RR (95%CI)	*P*	
Interventions in the control group								
Acupuncture	5	246	245	0	0.45	1.13 (1.07, 1.19)	<0.0001	Fixed
Western medicine	2	76	75	49	0.16	1.18 (1.05, 1.33)	0.006	Fixed
Other therapies	5	181	177	54	0.07	1.15 (1.03, 1.28)	0.01	Random
Index measurement time								
<15 days	5	176	172	56	0.06	1.14 (1.02, 1.27)	0.02	Random
≥15 days	7	327	325	0	0.45	1.15 (1.09, 1.21)	<0.00001	Fixed
Interventions in the treatment group								
Acupuncture combined with moxibustion	3	137	137	0	0.48	1.10 (1.03, 1.18)	0.007	Fixed
Acupuncture combined with other therapies	9	366	360	24	0.23	1.17 (1.11, 1.24)	<0.00001	Fixed
Mean duration								
>12 months	6	232	228	45	0.10	1.17 (1.10, 1.25)	<0.00001	Fixed
≤12 months	4	183	182	0	0.68	1.10 (1.04, 1.17)	0.002	Fixed
Not reported	2	88	87	0	0.62	1.22 (1.09, 1.37)	0.0008	Fixed
Mean age								
≥50 years old	4	157	156	0	0.70	1.21 (1.11, 1.32)	0.0003	Fixed
<50 years old	6	258	254	42	0.16	1.10 (1.04, 1.16)	<0.0001	Fixed
Not reported	2	88	87	0	0.62	1.22 (1.09, 1.37)	0.0008	Fixed

T: treatment group; C: control group.

### 4.3 NDI score

**Figure 5:**
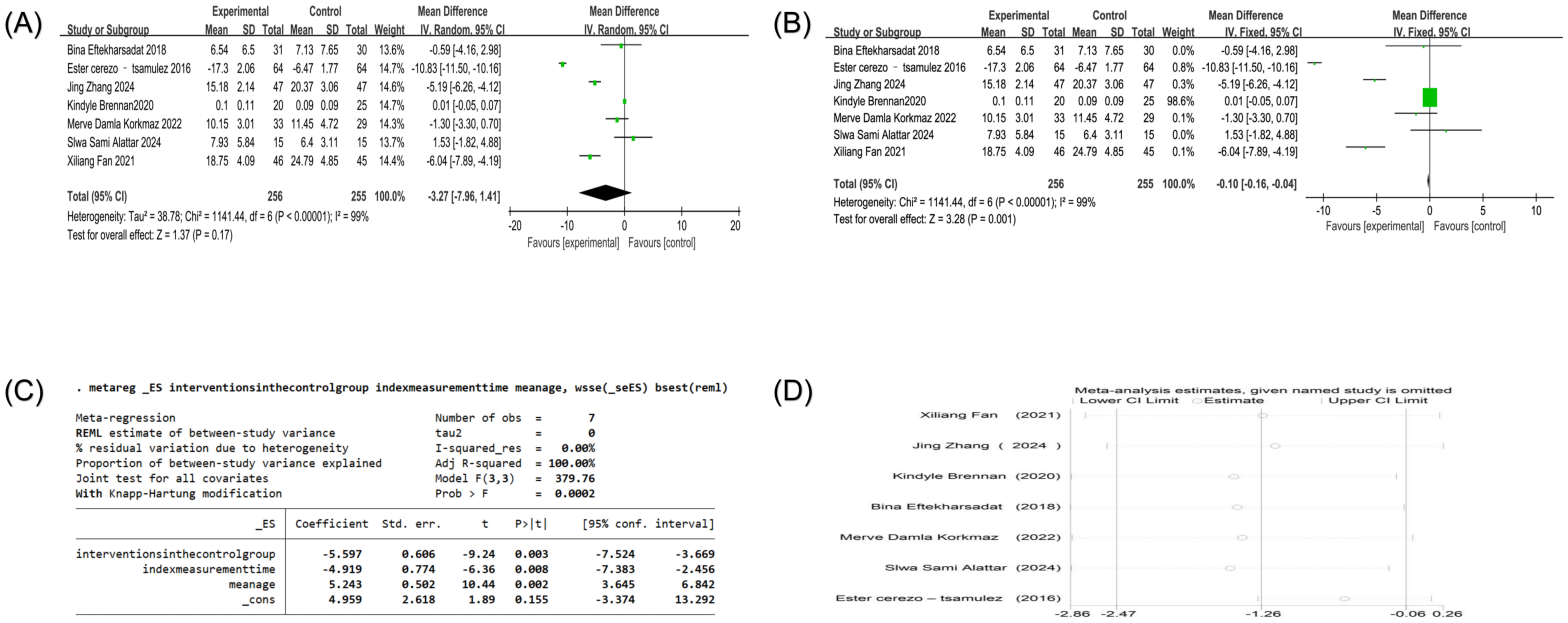
**(A)** Forest plot of NDI score of nape dorsal MPS treated by acupuncture combined therapy (random-effects model); **(B)** forest plot of NDI score of nape dorsal MPS treated by acupuncture combined therapy (fixed-effects model); **(C)** meta regression of NDI score of nape dorsal MPS treated with acupuncture combined therapy; **(D)** sensitivity analysis of NDI score in the treatment of nape dorsal MPS with acupuncture combined therapy.

A total of eight studies reported NDI scores, but due to the lack of data in one of the studies that met the measurement requirements, only seven studies were analyzed for NDI scores (4, 17, 19, 20, 26, 31, 35). Heterogeneity analysis showed that the heterogeneity was high (*p* < 0.00001, I^2^ = 99%) among the studies. The results of the random-effects model showed that there was no significant difference in the NDI score between the acupuncture combined group and the control group [MD = −3.27, 95%CI (−7.96, 1.41), Z = 1.37, *p* = 0.17]. Sensitivity analysis using a fixed-effects model yielded a significant result [MD = −0.1, 95%CI (−0.16, −0.04), *p* = 0.001], suggesting that the finding under the random-effects model is heavily influenced by a few studies with large effect sizes and precision (Figures 5A,B). Due to the high heterogeneity, meta-regression analysis, sensitivity analysis, and subgroup analysis were performed. The extreme heterogeneity (I^2^ = 99%) for the NDI score warrants careful consideration of its sources. Clinical diversity may stem from differences in the spectrum of cervical dysfunction severity at baseline, variations in the application of combined acupuncture therapies, and heterogeneity in adjunctive physical therapies or exercises. Methodological sources include potential variations in NDI questionnaire administration, cultural adaptation of the instrument, and study duration influencing functional recovery trajectories. Subgroup analysis showed that when the mean age was more than 35 years, the effect of the treatment group on improving the NDI score of patients with nape dorsal myofascial pain syndrome was significantly better than that of the control group [MD = −7.39, 95%CI (−11.57, −3.21), *p* = 0.0005]. There was no significant difference in NDI scores between the two groups when the mean age was 35 years or less [MD = 0.01, 95%CI (−0.05, 0.07), *p* = 0.76]. In addition, both are not affected by the different types of intervention measures in the control group and the measurement time of the indicators, which can improve the cervical spine dysfunction of the patients (Table 4). The results of the meta-regression analysis showed that the control group measures, measurement time, and mean age were significantly affected, indicating that the stricter the intervention in the control group, the lower the intervention effect in the treatment group; the longer the time of measuring the index, the intervention effect of acupuncture combined therapy may gradually weaken; and the effect of acupuncture combined therapy intervention on the mean age of the larger group is more significant (Figure 5C). The sensitivity analysis showed that the significance of the total effect depends on a few studies (4, 26). There was no significant change in heterogeneity, suggesting that the robustness of the combined results was low (Figure 5D).

**Table 4:** Subgroup analysis of NDI score of nape dorsal MPS treated with acupuncture combined therapy.

Subgroup	Research quality	Sample size		Heterogeneity analysis		Meta-analysis		Effects models
		T	C	I^2^ (%)	*P*	SMD/MD/RR (95%CI)	*P*	
Interventions in the control group								
Acupuncture	3	98	102	98	<0.00001	−2.00 (−6.11, 2.11)	0.34	Random
Other therapies	4	158	153	98	<0.00001	−4.29 (−9.91, 1.33)	0.13	Random
Index measurement time								
<30 days	3	94	89	90	<0.0001	−2.1 (−6.31, 2.11)	0.33	Random
≥30 days	4	162	166	100	<0.00001	−4.21 (−10.83, 2.42)	0.21	Random
Mean age								
>35 years old	3	157	156	98	<0.00001	−7.39 (−11.57, −3.21)	0.0005	Random
≤35 years old	4	99	99	0	0.47	0.01 (−0.05, 0.07)	0.76	Fixed

T: treatment group; C: control group.

### 4.4 Cervical function ROM score

**Figure 6:**
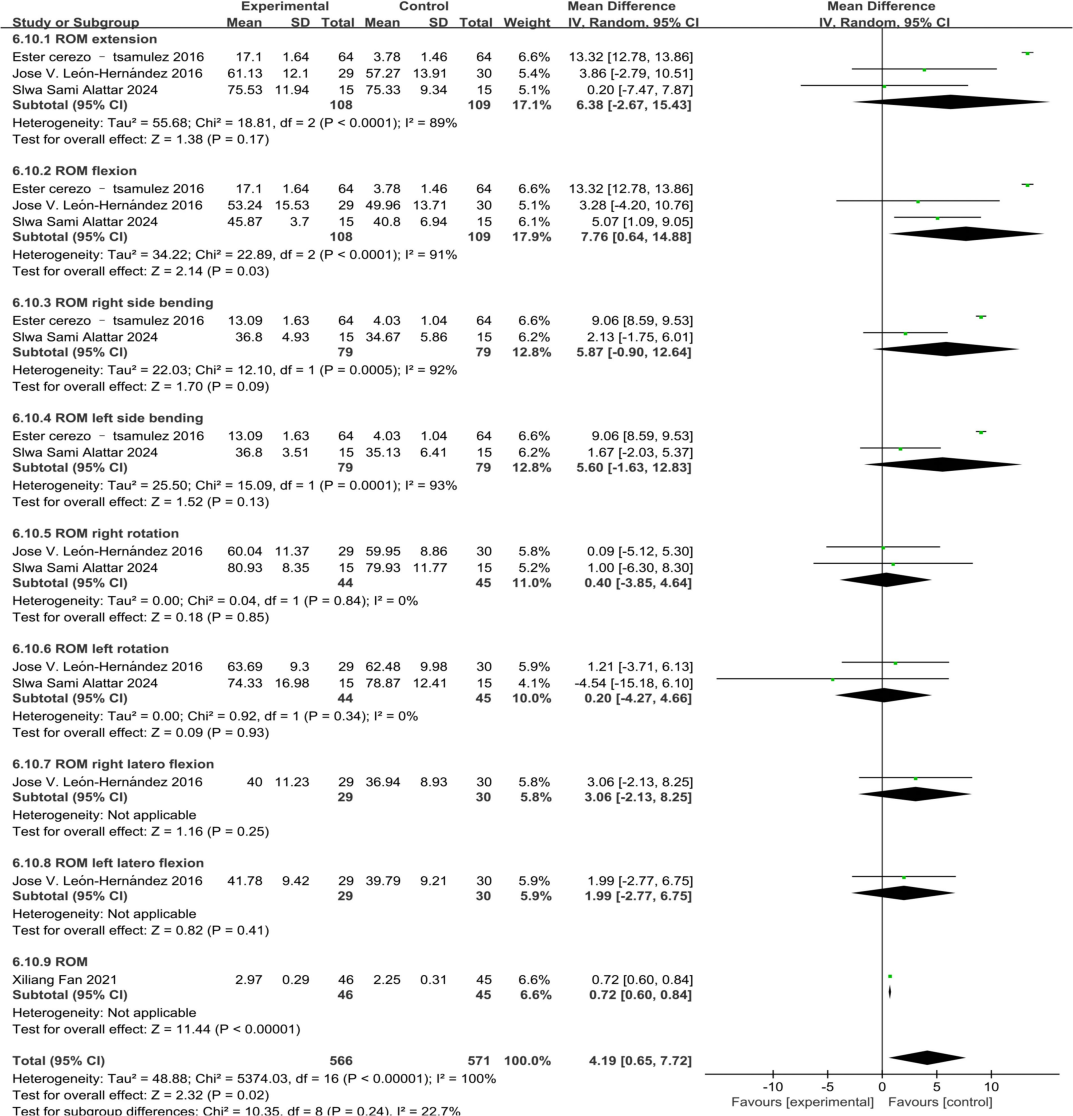
Forest map of cervical function ROM score of acupuncture combined with acupuncture in the treatment of neck dorsal MPS.

Four studies reported changes in cervical functional ROM, and three studies used a cervical goniometer to measure cervical function ROM score as an outcome measure (4, 20, 21). One study used the ROM score as an outcome indicator (31). Heterogeneity analysis showed that the heterogeneity of ROM extension, ROM flexion, ROM right side bending, and ROM left side bending was large (*P*<0.00001, I^2^ = 99%). Heterogeneity analysis of ROM right rotation and ROM left rotation suggested that studies were homogeneous (*p* = 0.85, I^2^ = 0%) (*p* = 0.35, I^2^ = 0%). Random--effects model analysis showed that the combined results of ROM extension, ROM right side bending, ROM left side bending, ROM right rotation, and ROM left rotation showed no significant difference between the treatment group and the control group. However, for ROM flexion, the effect of the treatment group was significantly better than that of the control group [MD = 7.76, 95%CI (0.64, 14.88), *p* = 0.03]. Only one study involved ROM right lateral flexion, ROM left lateral flexion, and ROM, so it was impossible to accurately evaluate the effect of acupuncture combined therapy on nape dorsal MPS (Figure 6).

### 4.5 PRI score

**Figure 7:**
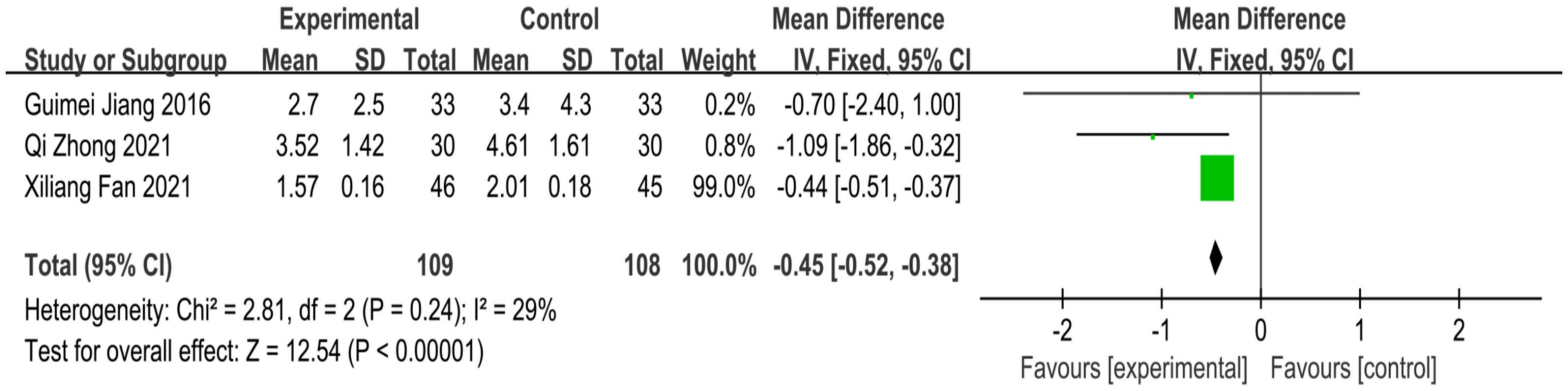
Forest plot of PRI score of nape dorsal MPS treated by acupuncture combined therapy.

Four studies reported PRI scores, but one of the studies’ data could not be converted, so only the PRI scores of three studies were analyzed (18, 23, 31). Heterogeneity analysis showed that the heterogeneity between the studies was low (*p* = 0.24, I^2^ = 29%). Fixed-effects model analysis showed that the treatment group could significantly reduce the PRI score compared with the control group [MD = −0.45, 95% CI (−0.52, −0.38), *P*<0.00001] (Figure 7).

### 4.6 PPI score

**Figure 8:**
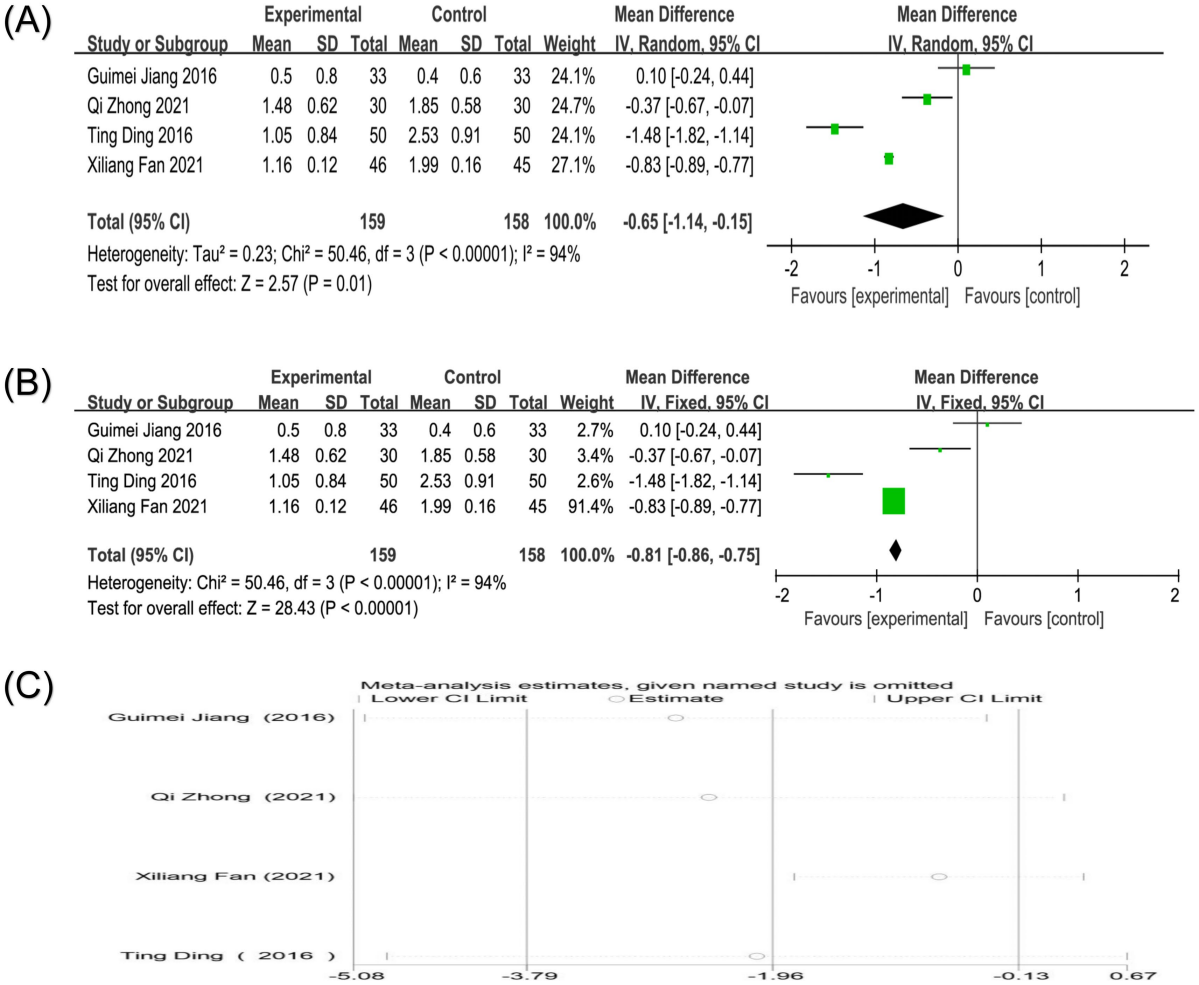
**(A)** Forest plot of PPI score of nape dorsal MPS treated by acupuncture combined therapy (random-effects model); **(B)** Forest plot of PPI score of nape dorsal MPS treated by acupuncture combined therapy (fixed-effects model); **(C)** Sensitivity analysis of PPI score in the treatment of nape dorsal MPS with acupuncture combined therapy.

Five studies reported PPI scores, one of which could not be converted, so only four studies were analyzed (18, 22, 23, 31). Heterogeneity analysis showed high heterogeneity among the studies (*p* < 0.00001, I^2^ = 94%). Random-effects model analysis showed that the treatment group could significantly reduce the effect of PRI score compared with the control group [MD = −0.65, 95%CI (−1.14, −0.15), *p* = 0.01]. A fixed-effects model sensitivity analysis produced a more precise result [MD = −0.81, 95%CI (−0.86, −0.75), *p* < 0.00001], reinforcing the conclusion that acupuncture combined therapy significantly reduces present pain intensity (Figures 8A,B). The high heterogeneity (I^2^ = 94%) for PPI score likely originates from clinical factors such as variations in the definition and measurement of “present pain,” the timing of assessment relative to treatment, and the baseline pain characteristics of the studied populations. Methodological contributions may include the use of different versions of pain inventories or subjective interpretation of the PPI scale categories. Sensitivity analysis showed that after excluding any single study, the results were still not significant, and the direction did not change fundamentally. The combined results maintained high robustness (Figure 8C).

### 4.7 SF-36 score

**Figure 9:**
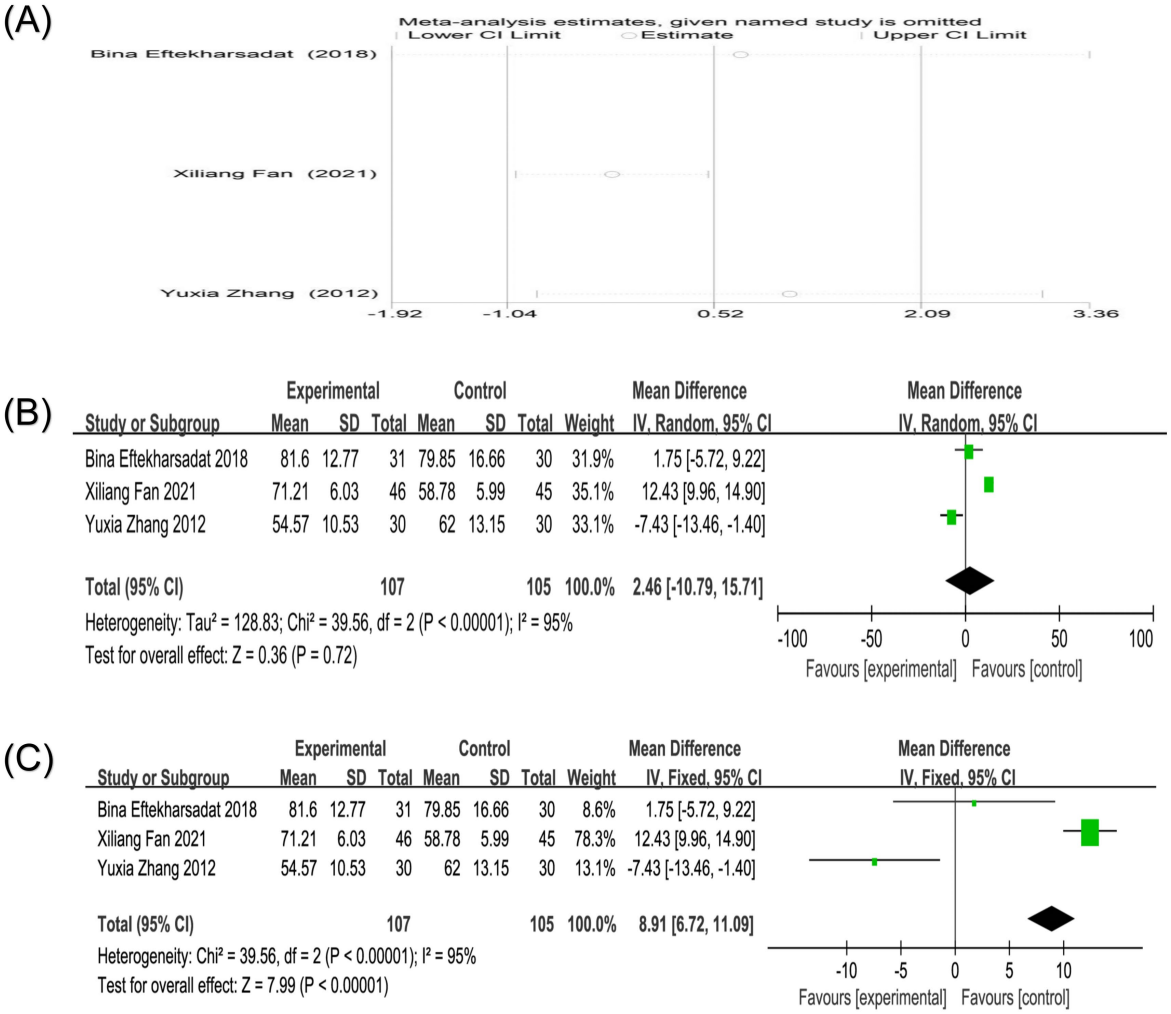
**(A)** Sensitivity analysis of SF-36 score in the treatment of nape dorsal MPS with acupuncture combined therapy; **(B)** Forest plot of SF-36 score of nape dorsal MPS treated by acupuncture combined therapy (random-effects model); **(C)** Forest plot of SF-36 score of nape dorsal MPS treated by acupuncture combined therapy (fixed-effects model).

Three studies reported the SF-36 score (17, 25, 31). Heterogeneity analysis showed high heterogeneity between the studies (*p* < 0.00001, I^2^ = 95%). Sensitivity analysis showed that after excluding any study, the combined results maintained high robustness (Figure 9A). Due to the limited number of studies and high heterogeneity, a random-effects model was used. Random-effects model analysis showed no significant difference between the two groups in reducing SF-36 scores [MD = 2.46, 95%CI (−10.79, 15.71), *p* = 0.72]. For comparison, a fixed-effects model was also applied, which showed a significant difference [MD = 8.91, 95%CI (6.72, 11.09), *p* < 0.00001], suggesting that the results of the random-effects model are influenced by a few studies with large effect sizes and that the conclusions cannot be definitively stated (Figures 9B,C). The extreme heterogeneity (I^2^ = 95%) for SF-36, despite only three studies, points to major differences in how the quality of life was impacted or measured. Clinical sources could include differing patient expectations, comorbidity profiles, or the specific life domains most affected by neck pain in each population. Methodologically, the use of different SF-36 versions or administration methods could be enhanced.

### 4.8 PPT score

**Figure 10:**
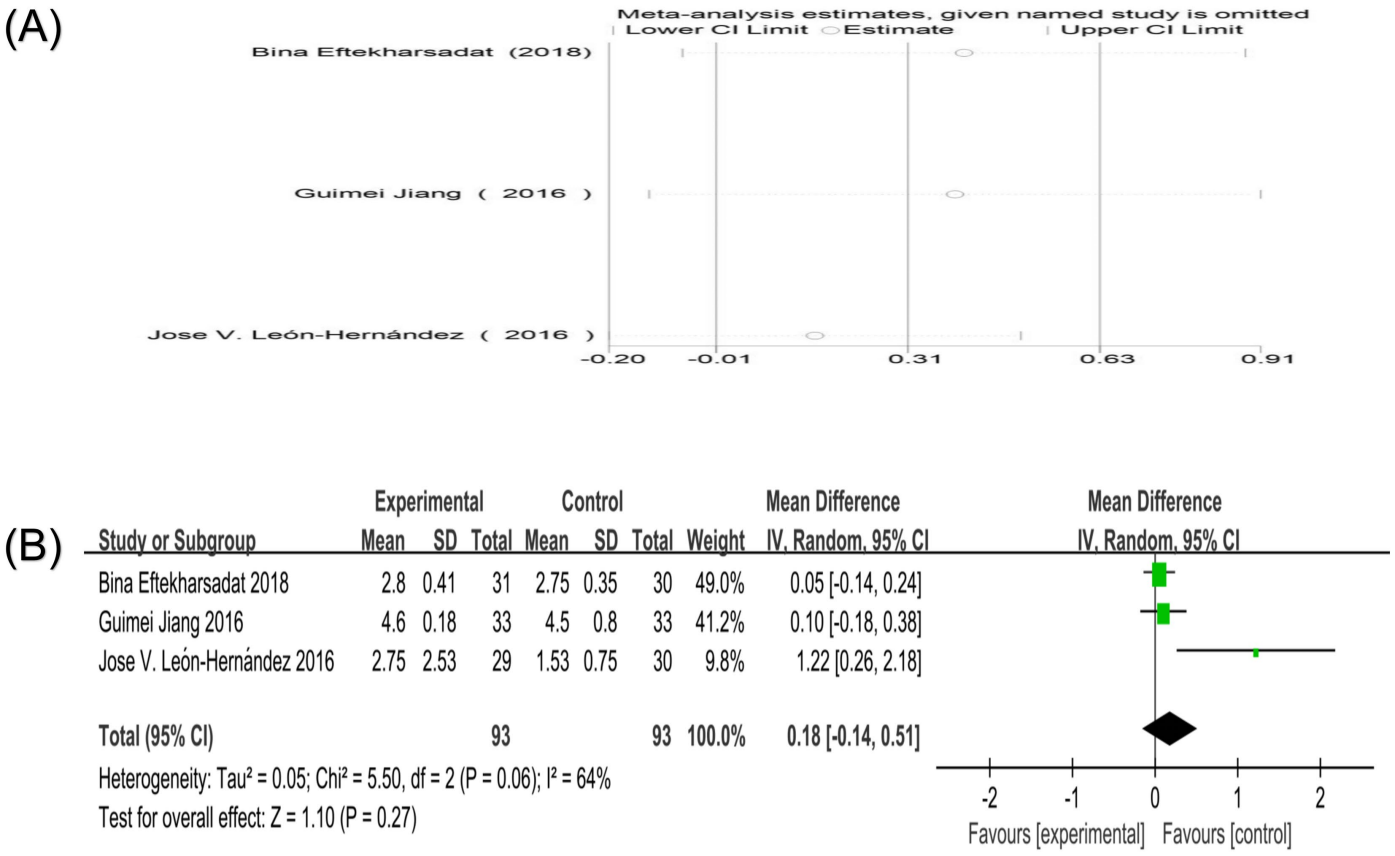
**(A)** Sensitivity analysis of PPT score in the treatment of nape dorsal MPS with acupuncture combined therapy; **(B)** Forest plot of PPT score of nape dorsal MPS treated by acupuncture combined therapy.

Four studies reported PPT scores (4, 17, 18, 21). Three studies used the overall PPT score as the outcome index (17, 18, 21). One study involved a single score of PPT in all directions (4). Heterogeneity analysis showed that the heterogeneity between studies was high (*p* = 0.06, I^2^ = 64%). Sensitivity analysis showed that after excluding any study, the combined results still maintained high robustness (Figure 10A). Random-effects model analysis showed no significant difference in PPT score between the two groups [MD = 0.18, 95%CI (−0.14, 0.51), *p* = 0.27] (Figure 10B). Since only one study included in the literature involved PPT right/left trapezius, PPT right/left levator, PPT right/left splenius, and PPT right/left multifidi, it is impossible to determine the therapeutic effect of acupuncture combined therapy on this index.

### 4.9 Oswestry score

**Figure 11:**
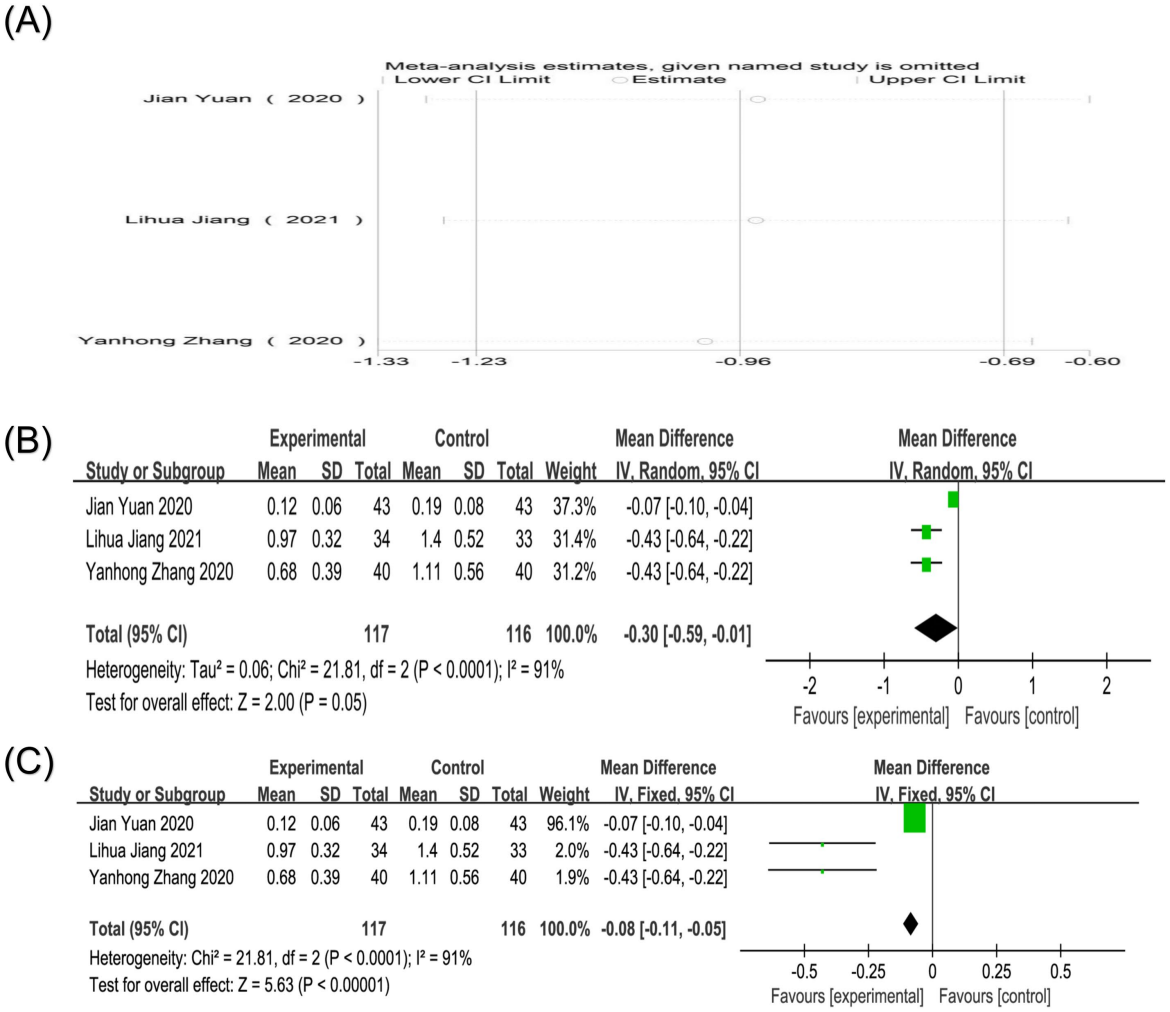
**(A)** Sensitivity analysis of Oswestry score in the treatment of nape dorsal MPS with acupuncture combined therapy; **(B)** Forest plot of Oswestry score of nape dorsal MPS treated by acupuncture combined therapy (random-effects model); **(C)** Forest plot of Oswestry score of nape dorsal MPS treated by acupuncture combined therapy (fixed-effects model).

Three studies reported Oswestry scores (27, 28, 30). Heterogeneity analysis showed that there was high heterogeneity among the studies (*p* < 0.0001, I^2^ = 91%). Sensitivity analysis showed that after excluding any study, the combined results maintained high robustness (Figure 11A). Random-effects model analysis showed that the effect of reducing Oswestry scores in the treatment group was slightly better than that in the control group [MD = −0.30, 95%CI (−0.59, −0.01), *p* = 0.05]. An analysis using a fixed-effects model yielded a nearly identical point estimate with a narrower confidence interval [MD = −0.08, 95%CI (−0.11, −0.05), *p* < 0.00001], strengthening the evidence for a modest but statistically significant benefit on disability (Figures 11B,C). The high heterogeneity (I2 = 91%) for the Oswestry score, used here presumably for cervical-related disability, may arise from differences in the primary location of myofascial pain, the specific functional limitations captured, and variations in the application of the Oswestry questionnaire for cervical conditions.

## 5 Publication bias

**Figure 12:**
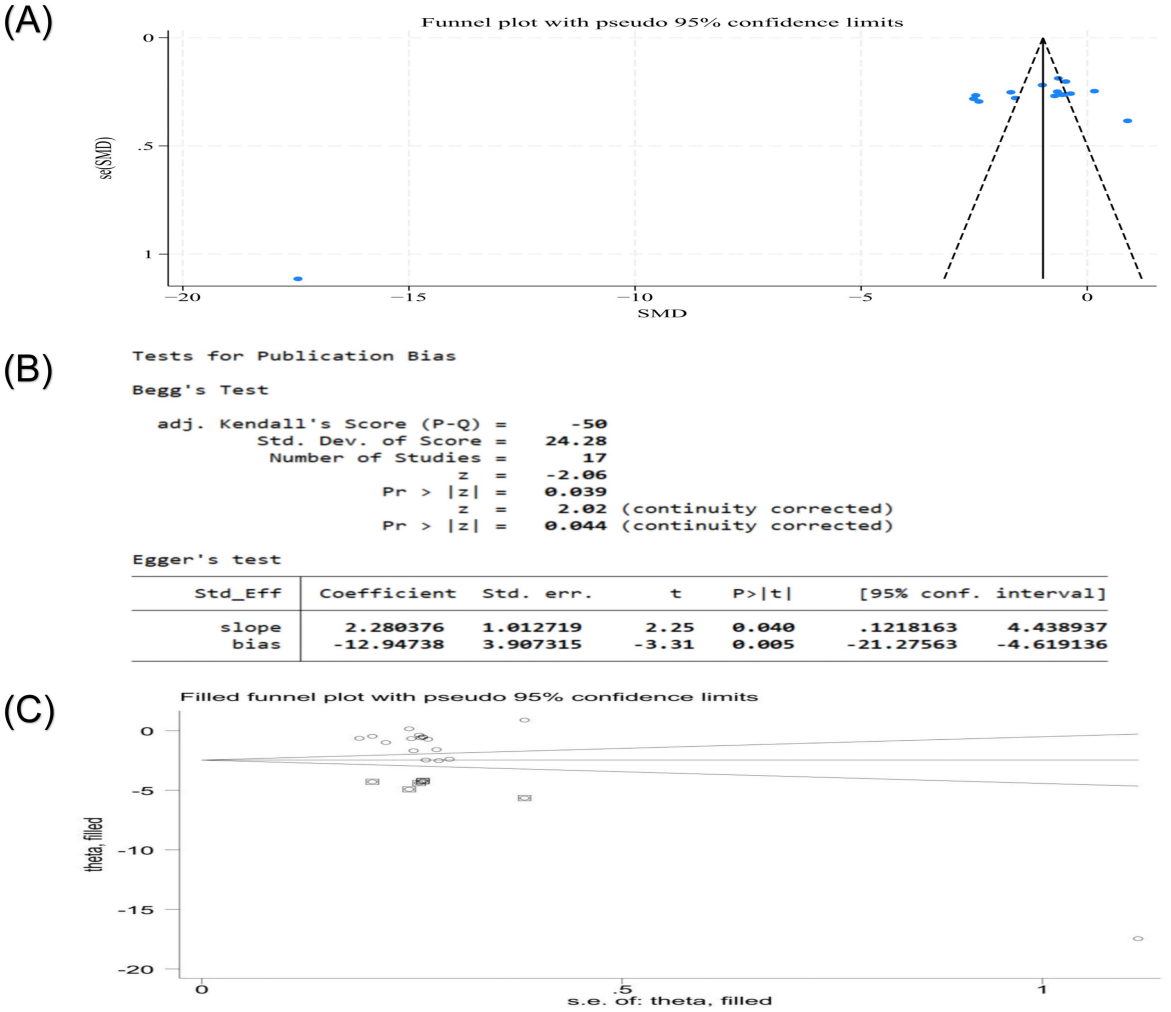
**(A)** Publication bias funnel plot of VAS score of nape dorsal MPS treated with acupuncture combined therapy; **(B)** Egger ‘s test and Begg ‘s test of VAS score of nape dorsal MPS treated with acupuncture combined therapy; **(C)** Publication bias trim funnel plot of VAS score of nape dorsal MPS treated by acupuncture combined therapy.

**Figure 13:**
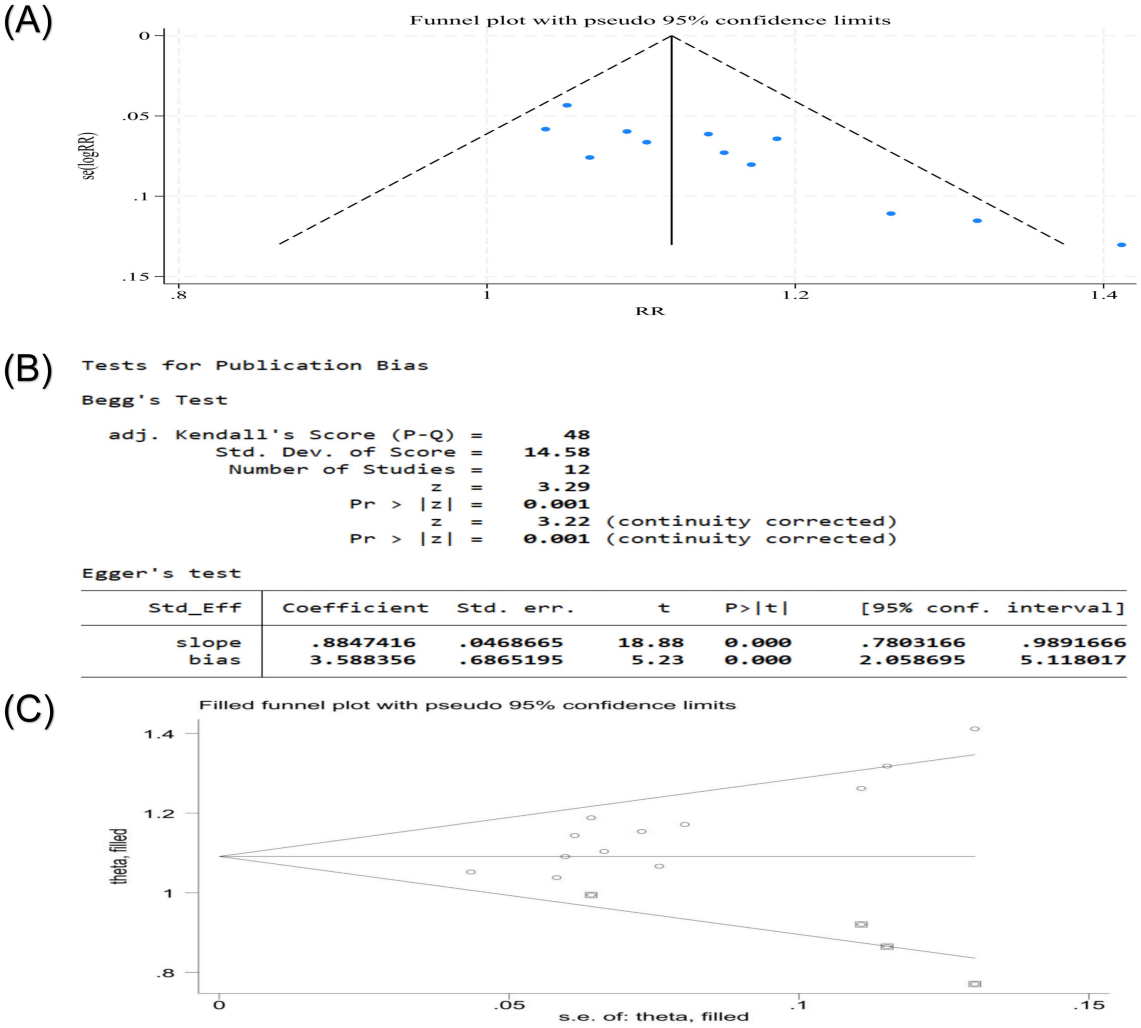
**(A)** Publication bias funnel plot of clinical effective rate of acupuncture combined therapy in the treatment of nape dorsal MPS; **(B)** Egger ‘s test and Begg ‘s test of the clinical effective rate of acupuncture combined with acupuncture in the treatment of nape dorsal MPS; **(C)** Publication bias trim funnel plot of clinical effective rate of acupuncture combined therapy in the treatment of nape dorsal MPS.

Publication bias was assessed for the VAS score and clinical effective rate, the only outcomes with sufficient studies (*n* > 10). The VAS score showed significant funnel plot asymmetry, and the results of Egger’s test and Begg’s test were *p* < 0.05, suggesting that there may be publication bias in the VAS score (Figures 12A,B). To address potential missing studies, the trim-and-fill method was applied, which iteratively imputes theoretically missing studies to achieve funnel plot symmetry. After four iterations, six missing literatures were estimated. The results showed that the heterogeneity before trim-and-fill methods (Q = 385.525, *p* = 0.000) and after (Q = 1236.210, *p* = 0.000) was high. The adjusted effect size [standard error (SMD) = 0.085, 95%CI (0.037, 0.195)] lost statistical significance, indicating the result was not robust (Figure 12C). For the clinical effective rate, similar asymmetry and statistical tests suggested potential bias (Figures 13A,B). The heterogeneity, which was low before adjustment (Q = 16.758, *p* = 0.115), after imputing four missing studies, increased after imputation (Q = 33.449, *p* = 0.004), and the adjusted estimate [logRR = 2.992, 95%CI (2.828, 3.165)] maintained statistical significance and direction of the finding, demonstrating result robustness despite potential publication bias (Figure 13C).

## 6 Safety evaluation

A comprehensive safety assessment based on the included studies showed that of the 21 randomized controlled trials, only 5 (4, 18, 19, 26, 35) explicitly reported safety monitoring results, with 1 study reporting 1 case of an allergic reaction to acupuncture leading to discontinuation of treatment (19) and 4 studies explicitly documenting that no adverse events occurred (4, 18, 26, 35), and the remaining 16 studies did not mention safety monitoring information (17, 20–25, 27–34, 36) (Table 5). The available evidence suggests that fewer and less severe adverse events are reported with acupuncture combination therapy in the treatment of nape dorsal MPS, but current evidence is insufficient to establish a complete safety profile due to methodological limitations such as small sample sizes, short follow-up times, and a lack of systematic reporting of safety in the majority of studies. A systematic monitoring mechanism for adverse events should be established in clinical practice, and larger, methodologically rigorous prospective studies with standardized reporting protocols should be conducted to assess the safety of this therapy accurately.

**Table 5:** Summary of adverse events.

Adverse event/number of event	Allergic	Headache	Vomit	Other adverse reactions
Jing (33)	—	—	—	—
Wen-sheng et al (32)	—	—	—	—
Xi-liang (31)	—	—	—	—
Feng et al. (26)	—	—	—	—
Yan-hong et al. (27)	—	—	—	—
Jing et al. (26)	no/0	no/0	no/0	no/0
Zhi-ping (34)	—	—	—	—
Bao-guo et al. (36)	—	—	—	—
Yu-xia (25)	—	—	—	—
Le-qin (24)	—	—	—	—
Qi (23)	—	—	—	—
Ting (22)	—	—	—	—
Li-hua (28)	—	—	—	—
Jian and Hua (30)	—	—	—	—
Brennan et al. (35)	no/0	no/0	no/0	no/0
Leon-Hernandez et al. (21)	—	—	—	—
Eftekharsadat et al. (17)	—	—	—	—
Korkmaz et al. (19)	yes/1	no/0	no/0	no/0
Sami Alattar and Alzahrani (20)	—	—	—	—
Jiang et al. (18)	no/0	no/0	no/0	no/0
Cerezo-Téllez et al. (4)	no/0	no/0	no/0	no/0

“—”: indicates that the study did not mention or report safety monitoring or adverse events; “no/0” indicates the study explicitly reported no adverse events for that category; “yes/1” indicates the study documented one adverse event.

## 7 Evidence quality assessment

GRADE evidence evaluation showed that intermediate evidence supported that acupuncture combined therapy could significantly improve the total clinical effective rate, PRI score, tenderness score, rest pain efficacy, PPT right/left trapezius, and neck muscle strength compared with other clinical routine therapies. Low-level evidence supports that acupuncture combined therapy can improve the Cervical Range of Motion (CROM); score, ROM right/left rotation, ROM right/left lateral flexion, Pittsburgh Sleep Quality Index (PSQI); score, IL-1*β* (Interleukin-1β); content, COX-2 (Cyclooxygenase-2); Tumor Necrosis Factor (TNF-*α*); content, 25(OH)D content, β-EP (β-Endorphin); content, 6-keto-PGE1 (Prostaglandin E1); content, SP content, 5-HT (5-Hydroxytryptamine); content, fascia thickness, tissue elasticity score, Young’s modulus value, average amplitude value, average frequency slope, GQOLI-74 (Generic Quality of Life Inventory-74); score, other symptom improvement efficacy, Roland–Morris score tenderness score, recovery time, NPRS (Numerical Pain Rating Scale); score, PPT (Pressure Pain Threshold); right/left levator, PPT (Pressure Pain Threshold); right/left splenius, PPT (Pressure Pain Threshold); right/left multifidi, PNS (Peripheral Nervous System); thickness of UT (Upper Trapezius); muscle, diameter of the MTrP (Myofascial Trigger Point); and BDI (Beck Depression Inventory) scores of patients with nape dorsal MPS compared with clinical routine therapy. Other outcome indicators, including the VAS score, NDI score, SF-36 score, and PPT score, were assessed as very low-quality evidence. These ratings stemmed from serious concerns about inconsistency (extreme heterogeneity), high risk of publication bias (as evidenced by funnel plot asymmetry and statistical tests), and imprecision. The high degree of heterogeneity observed in these results could not be fully explained by subgroup analyses or meta-regression analyses, suggesting that the available variables did not capture the underlying clinical or methodological diversity (Table 6).

**Table 6:** Evidence quality evaluation table of outcome indicators of acupuncture combined therapy for nape dorsal MPS.

Outcome index	Sample size T/C	Quality audit					RR/MD/SMD (95%CI)	Quality rating
		Risk of bias	Discordance	Indirectness	Inaccuracy	Publication bias		
VAS score	668/665	Downgrade^a^	Lower level 2^b^	No downgrade	No downgrade	No downgrade	MD = −1.51, 95%CI (−2.12, −0.90)	Very low
clinical effective rates	503/47	Downgrade^a^	No downgrade	No downgrade	No downgrade	No downgrade	RR = 1.15, 95%CI (1.10, 1.20)	Medium
NDI score	256/255	Downgrade^a^	Lower level 2^b^	No downgrade	Downgrade^d^	No downgrade	MD = −3.27, 95%CI (−7.96, 1.41)	Very low
Cervical function ROM score	46/45	Downgrade^a^	No downgrade	No downgrade	Downgrade^d^	No downgrade	MD = 0.72, 95%CI (0.60, 0.84)	Low
ROM extension	108/109	Downgrade^a^	Lower level 2^b^	No downgrade	Downgrade^d^	No downgrade	MD = 6.38, 95%CI (−2.67, 15.43)	Very low
ROM flexion	108/109	Downgrade^a^	Lower level 2^b^	No downgrade	No downgrade	No downgrade	MD = 7.76, 95%CI (0.64, 14.88)	Very low
ROM right side bending	79/79	Downgrade^a^	Lower level 2^b^	No downgrade	Downgrade^d^	No downgrade	MD = 5.87, 95%CI (−0.9, 12.64)	Very low
ROM left side bending	79/79	Downgrade^a^	Lower level 2^b^	No downgrade	Downgrade^d^	No downgrade	MD = 5.60, 95%CI (−1.63, 12.83)	Very low
ROM right rotation	44/45	Downgrade^a^	No downgrade	No downgrade	Downgrade^d^	No downgrade	MD = 0.40, 95%CI (−3.85, 4.64)	Low
ROM left rotation	44/45	Downgrade^a^	No downgrade	No downgrade	Downgrade^d^	No downgrade	MD = 0.20, 95%CI (−4.27, 4.66)	Low
ROM right latero flexion	29/30	Downgrade^a^	No downgrade	No downgrade	Downgrade^d^	No downgrade	MD = 3.06, 95%CI (−2.13, 8.25)	Low
ROM left latero flexion	29/30	Downgrade^a^	No downgrade	No downgrade	Downgrade^d^	No downgrade	MD = 1.99, 95%CI (−2.77, 6.75)	Low
PRI score	109/108	Downgrade^a^	No downgrade	No downgrade	No downgrade	No downgrade	MD = −0.45, 95%CI (−0.52, −0.38)	Medium
PPI score	109/108	Downgrade^a^	Lower level 2^b^	No downgrade	Downgrade^d^	No downgrade	MD = −0.39, 95%CI (−0.95, 0.18)	Very low
SF-36 score	107/105	Downgrade^a^	Lower level 2^b^	No downgrade	Downgrade^d^	No downgrade	MD = 2.46, 95%CI (−10.79, 15.71)	Very low
PSQI score	46/45	Downgrade^a^	No downgrade	No downgrade	Downgrade^d^	No downgrade	MD = −5.56, 95%CI (−6.17, −4.95)	Low
IL-1β content	47/47	Downgrade^a^	No downgrade	No downgrade	Downgrade^d^	No downgrade	MD = −1.05, 95%CI (−1.45, −0.65)	Low
COX − 2 content	47/47	Downgrade^a^	No downgrade	No downgrade	Downgrade^d^	No downgrade	MD = −9.45, 95%CI (−12.53, −6.37)	Low
TNF-α content	47/47	Downgrade^a^	No downgrade	No downgrade	Downgrade^d^	No downgrade	MD = −26.52, 95%CI (−34.76, −18.28)	Low
25(OH)D content	47/47	Downgrade^a^	No downgrade	No downgrade	Downgrade^d^	No downgrade	MD = 12.13, 95%CI (6.84, 17.42)	Low
β-EP content	47/47	Downgrade^a^	No downgrade	No downgrade	Downgrade^d^	No downgrade	MD = 39.85, 95%CI (30.25, 49.45)	Low
6-keto-PGE1 content	47/47	Downgrade^a^	No downgrade	No downgrade	Downgrade^d^	Nossss downgrade	MD = −7.47, 95%CI (−10.07, −4.87)	Low
SP content	47/47	Downgrade^a^	No downgrade	No downgrade	Downgrade^d^	No downgrade	MD = −48.47, 95%CI (−59.77, −37.17)	Low
5-HT content	47/47	Downgrade^a^	No downgrade	No downgrade	Downgrade^d^	No downgrade	MD = −9.05, 95%CI (−11.56, −6.54)	Low
Fascia thickness	47/47	Downgrade^a^	No downgrade	No downgrade	Downgrade^d^	No downgrade	MD = −1.27, 95%CI (−1.44, −1.10)	Low
Tissue elasticity map score	47/47	Downgrade^a^	No downgrade	No downgrade	Downgrade级^d^	No downgrade	MD = −0.65, 95%CI (−0.87, −0.43)	Low
young’s modulus value	47/47	Downgrade^a^	No downgrade	No downgrade	Downgrade^d^	No downgrade	MD = −7.67, 95%CI (−9.59, −5.75)	Low
Average amplitude value	47/47	Downgrade^a^	No downgrade	No downgrade	Downgrade^d^	No downgrade	MD = 3.81, 95%CI (2.65, 4.97)	Low
Average frequency slope	47/47	Downgrade^a^	No downgrade	No downgrade	Downgrade^d^	No downgrade	MD = 0.01, 95%CI (0.01, 0.01)	Low
GQOLI-74 score	47/47	Downgrade^a^	No downgrade	No downgrade	Downgrade^d^	No downgrade	MD = 21.35, 95%CI (5.74, 36.96)	Low
Rest pain effect	60/55	Downgrade^a^	No downgrade	No downgrade	No downgrade	No downgrade	RR = 1.22, 95%CI (1.04, 1.44)	Medium
Improvement of other symptoms	60/55	Downgrade^a^	No downgrade	No downgrade	Downgrade^d^	No downgrade	RR = 1.18, 95%CI (0.99, 1.40)	Low
Roland-Morris score	30/30	Downgrade^a^	No downgrade	No downgrade	Downgrade^d^	No downgrade	MD = −1.83, 95%CI (−3.61, −0.05)	Low
pressure pain score	50/50	Downgrade^a^	No downgrade	No downgrade	No downgrade	No downgrade	MD = −0.61, 95%CI (−0.64, −0.58)	Medium
Oswestry score	117/116	Downgrade^a^	Lower Level 2^b^	No downgrade	No downgrade	No downgrade	MD = −0.30, 95%CI (−0.59, −0.01)	Very low
The time taken to heal	43/43	Downgrade^a^	No downgrade	No downgrade	Downgrade^d^	No downgrade	MD = −4.64, 95%CI (−6.77, −2.51)	Low
NPRS	20/25	Downgrade^a^	No downgrade	No downgrade	Downgrade^d^	No downgrade	MD = 0.56, 95%CI (−0.42, 1.54)	Low
PPT score	93/93	Downgrade^a^	Downgrade^c^	No downgrade	Downgrade^d^	No downgrade	MD = 0.18, 95%CI (−0.14, 0.51)	Very low
PPT right trapezius	59/54	Downgrade^a^	No downgrade	No downgrade	No downgrade	No downgrade	MD = 1.82, 95%CI (1.75, 1.89)	Medium
PPT left trapezius	54/53	Downgrade^a^	No downgrade	No downgrade	No downgrade	No downgrade	MD = 1.69, 95%CI (1.62, 1.76)	Medium
PPT right levator	40/39	Downgrade^a^	No downgrade	No downgrade	Downgrade^d^	No downgrade	MD = 1.95, 95%CI (1.84, 2.06)	Low
PPT left levator	25/35	Downgrade^a^	No downgrade	No downgrade	Downgrade^d^	No downgrade	MD = 2.69, 95%CI (2.51, 2.87)	Low
PPT right splenius	24/36	Downgrade^a^	No downgrade	No downgrade	Downgrade^d^	No downgrade	MD = 1.03, 95%CI (0.92, 1.1.14)	Low
PPT left splenius	24/36	Downgrade^a^	No downgrade	No downgrade	Downgrade^d^	No downgrade	MD = 1.64, 95%CI (1.46, 1.82)	Low
PPT right multifidi	41/48	Downgrade^a^	No downgrade	No downgrade	Downgrade^d^	No downgrade	MD = 2.17, 95%CI (2.08, 2.26)	Low
PPT left multifidi	32/45	Downgrade^a^	No downgrade	No downgrade	Downgrade^d^	No downgrade	MD = 2.17, 95%CI (2.06, 2.28)	Low
PNS	29/30	Downgrade^a^	No downgrade	No downgrade	Downgrade^d^	No downgrade	MD = −2.03, 95%CI (−3.53, −0.52)	Low
Thickness of UT muscle	33/29	Downgrade^a^	No downgrade	No downgrade	Downgrade^d^	No downgrade	MD = −0.24, 95%CI (−0.98, 0.50)	Low
Diameter of the MTrP	33/29	Downgrade^a^	No downgrade	No downgrade	Downgrade^d^	No downgrade	MD = −1.14, 95%CI (−1.92, −0.36)	Low
BDI	15/15	Downgrade^a^	No downgrade	No downgrade	Downgrade^d^	No downgrade	MD = 1.26, 95%CI (−4.89, 7.41)	Low
Neck muscles strength (right rotation)	64/64	Downgrade^a^	No downgrade	No downgrade	No downgrade	No downgrade	MD = 20.96, 95%CI (20.25, 21.67)	Medium
Neck muscles strength (left rotation)	64/64	Downgrade^a^	No downgrade	No downgrade	No downgrade	No downgrade	MD = 24.3, 95%CI (23.57, 25.03)	Medium
Neck muscles strength (right side bending)	64/64	Downgrade^a^	No downgrade	No downgrade	No downgrade	No downgrade	MD = 21.31, 95%CI (20.43, 22.19)	Medium
Neck muscles strength (left side bending)	64/64	Downgrade^a^	No downgrade	No downgrade	No downgrade	No downgrade	MD = 21.31, 95%CI (20.40, 22.22)	Medium
Neck muscles strength (flexion)	64/64	Downgrade^a^	No downgrade	No downgrade	No downgrade	No downgrade	MD = 26.31, 95%CI (25.38, 27.24)	Medium
Neck muscles strength (extension)	64/64	Downgrade^a^	No downgrade	No downgrade	No downgrade	No downgrade	MD = 27.92, 95%CI (26.88, 28.96)	Medium

a Lack of specific details of allocation concealment and blinding.

b I^2^ value > 75%, indicating that there is a very serious inconsistency.

c 75% ≥ I^2^ > 50%, indicating that there is a serious inconsistency.

d The sample size included in the study was small, the confidence interval was wide, and the invalid line was crossed.

## 8 Discussion

This meta-analysis and systematic evaluation of 21 studies demonstrated that patients receiving acupuncture combined therapy showed significantly greater improvements in VAS score, clinical effective rate, cervical ROM flexion, PRI score, and Oswestry score compared to control interventions. These findings support the potential clinical benefit of integrating multiple acupuncture-based modalities in the management of nape dorsal MPS.

The GRADE assessment indicates that while primary outcomes are supported by moderate-certainty evidence, the overall conclusions are constrained by the limited quality of several secondary outcomes. Specifically, the moderate certainty for the clinical effective rate and PRI score enhances confidence in these findings, whereas the very low certainty for VAS and NDI scores necessitates cautious interpretation despite their statistical significance. This disparity highlights that while acupuncture combined therapy demonstrates consistent benefits across multiple domains, the strength of evidence varies substantially depending on the outcome metric.

Sensitivity analyses confirmed the robustness of the majority of outcomes, though they also identified key sources of high heterogeneity in several measures. Although the subgroup analysis showed a slight change in the heterogeneity and combined effect of some results, the overall findings remained robust. Meta-regression and subgroup analyses further revealed potential effect modifiers—notably, the stronger effects observed in patients over 35 years of age for both VAS and NDI outcomes suggest that age may be an essential moderator, possibly reflecting age-related differences in pathophysiology or treatment response.

Heterogeneity analysis showed that heterogeneity was low for outcomes such as the clinical effective rate and PRI score, supporting the reliability of the results, whereas extreme between-study variation (I^2^ > 90%) was observed for VAS, NDI, PPI, SF-36, and Oswestry scores. Although subgroup analyses yielded slight reductions in heterogeneity for some outcomes, they did not fully account for the observed variance, yet the overall findings remained robust. Meta-regression and subgroup analyses further revealed potential effect modifiers. Notably, the stronger effects observed in patients over 35 years of age for both VAS and NDI outcomes suggest that age may be an essential moderator, possibly reflecting age-related differences in pathophysiology or treatment response. While the meta-analysis provides evidence of a beneficial effect on average, its applicability to specific clinical contexts requires careful consideration of these potential effect modifiers.

Publication bias analyses suggest a potential risk of publication bias for VAS scores and clinical effectiveness. The use of the trim-and-fill method provided insights into the potential impact of publication bias. VAS results are particularly susceptible to bias, as statistical adjustments can substantially alter the effect estimate and nullify its statistical significance, thereby weakening the robustness of the original finding. In contrast, clinical validity results remain stable after adjustment. Funnel plot asymmetry, along with significant Egger’s and Begg’s tests, suggests possible omission of smaller negative studies.

Limitations of this study: First, the limited number of studies, small sample size, and generally low methodological quality increased the risk of publication bias; second, variability in study design (including control interventions, treatment duration, acupuncture protocols, and patient characteristics) introduced potential bias, which was exacerbated by allocation concealment and inadequate blinding; third, pain-related outcomes are vulnerable to publication bias, and conclusions are unstable; fourth, the majority of studies lacked long-term follow-up, preventing the assessment of sustained effects; fifth, included studies predominantly from Asian populations and Chinese databases, and lack of comprehensiveness in ethnicity and population; sixth, language bias may exist as only Chinese and English databases were searched; seventh, inadequate reporting of adverse events and safety profiles prevents a comprehensive risk–benefit analysis; and eighth, the definition of “acupuncture combination therapy”is overly broad, encompassing an excessive number of acupuncture combination regimens with vastly differing mechanisms of action, resulting in confounding effect sizes. Subgroup sample sizes were too small to draw definitive conclusions regarding specific combination regimens.

Acupuncture combined therapy shows potential short-term efficacy for nape dorsal MPS. However, considerable heterogeneity, potential publication bias, geographic limitations, and insufficient safety data warrant caution in interpreting the findings and preclude definitive conclusions. Future research should prioritize high-quality, multicenter RCTs that incorporate the following: (1) standardized protocols with detailed descriptions of acupuncture techniques and combination therapies; (2) implementation of rigorous methodology, including adequate allocation concealment and blinded outcome assessment where possible; (3) longer follow-up periods to evaluate the sustainability of treatment effects; (4) enrollment of diverse populations to enhance the generalizability of findings; and (5) proactive monitoring and comprehensive reporting of adverse events to establish a reliable safety profile.

## Data Availability

The original contributions presented in the study are included in the article/supplementary material, further inquiries can be directed to the corresponding author.
